# Meprin β metalloproteases associated with differential metabolite profiles in the plasma and urine of mice with type 1 diabetes and diabetic nephropathy

**DOI:** 10.1186/s12882-019-1313-2

**Published:** 2019-04-25

**Authors:** Jessica Gooding, Lei Cao, Courtney Whitaker, Jean-Marie Mwiza, Mizpha Fernander, Faihaa Ahmed, Zach Acuff, Susan McRitchie, Susan Sumner, Elimelda Moige Ongeri

**Affiliations:** 1 NIH Eastern Regional Comprehensive Metabolomics Resource Core, University of North Carolina, Chapel Hill, NC USA; 2 Discovery Science & Technology, RTI International, Research Triangle Park, NC USA; 3 Department of Biology, North Carolina A&T State University, Greensboro, NC 27411 USA; 4 Nutrition Research Institute, University of North Carolina, Chapel Hill, NC USA

**Keywords:** Meprin β metalloprotease, Diabetic kidney injury, Metabolomics, Metabolites

## Abstract

**Background:**

Meprin metalloproteases are abundantly expressed in the brush border membranes of kidney proximal tubules and small intestines. Meprins are also expressed in podocytes and leukocytes (monocytes and macrophages). Meprins are implicated in the pathophysiology of diabetic nephropathy (DN) but underlying mechanisms are not fully understood. Single nucleotide polymophisms (SNPs) in the meprin β gene were associated with DKD in human subjects. Furthermore, meprin α and β double deficiency resulted in more severe kidney injury and higher mortality rates in mice with Streptozotocin (STZ)-induced type 1 diabetes. Identification of meprin substrates has provided insights on how meprins could modulate kidney injury. Meprin targets in the kidney include extracellular matrix (ECM) proteins, modulators of inflammation, and proteins involved in the protein kinase A (PKA) and PKC signaling pathways. The current study used a global metabolomics approach to determine how meprin β expression impacts the metabolite milieu in diabetes and DKD.

**Methods:**

Low dose STZ was used to induce type 1 diabetes in 8-week old wild-type (WT) and meprin β knockout (βKO) mice. Blood and urine samples were obtained at 4 and 8 weeks post-STZ injection. Assays for albumin, creatinine, neutrophil gelatinase-associated lipocalin (NGAL), kidney injury molecule − 1 (KIM-1), and cystatin C were used for biochemical assessment of kidney injury. Data for biomarkers of kidney injury utilized two-way ANOVA. Metabolomics data analysis utilized UPLC-QTOF MS and multivariate statistics.

**Results:**

The number of metabolites with diabetes-associated changes in levels were significantly higher in the WT mice when compared to meprin βKO counterparts. Annotated meprin β expression-associated metabolites with strong variable importance in projection (VIP) scores play roles in lipid metabolism (LysoPC(16:1(9Z)), taurocholic acid), amino acid metabolism (indoxyl sulfate, hippuric acid), and neurotransmitter/stress hormone synthesis (cortisol, 3-methoxy-4-hydroxyphenylethylene glycolsulfate, homovanillic acid sulfate). Metabolites that associated with meprin β deficiency include; 3,5-dihydroxy-3′,4′-dimethoxy-6,7-methylenedioxyflavone 3-glucuronide, pantothenic acid, and indoxyl glucuronide (all decreased in plasma).

**Conclusion:**

Taken together, the annotated metabolites suggest that meprin β impacts complications of diabetes such as DKD by altering distinct metabolite profiles.

**Electronic supplementary material:**

The online version of this article (10.1186/s12882-019-1313-2) contains supplementary material, which is available to authorized users.

## Background

Diabetes is the leading cause of chronic kidney disease (CKD) with 20–40% of patients with type 2 diabetes (T2D) developing diabetic nephropathy (DN). For patients at risk for progressing to DN, early diagnosis and targeted interventions are hindered by the lack of sensitive and accurate tools for early diagnosis. This is in part because the underlying cellular and molecular mechanisms are not fully understood. Meprins are zinc metalloproteases of the astacin family and are most abundantly expressed in the brush border membranes (BBM) of proximal kidney tubules and small intestines [1, 2]. Meprins are also expressed in podocytes [3], and leukocytes (monocytes and macrophages) [4]. Meprins are composed of α and β subunits, that are encoded by distinct genes, resulting in two highly similar protein isoforms [5, 6]. Meprin A is a homooligomer of α subunits (α-α) or a heterodimer of α and β subunits (α-β), while meprin B is a homooligomer of β subunits (β-β) [7]. Meprins have been implicated in the pathology of DN in human patients and in mouse models of DN [8–11]. Meprin β gene single nucleotide polymorphisms (SNPs) were associated with DN and end stage renal disease (ESRD) in the Pima Indians, a Native American ethnic group with extremely high incidences of type 2 diabetes and ESRD [9]. Both meprin α and meprin β gene and protein expression levels were down-regulated in the kidneys of diabetic rats, and db/db mice before development of overt kidney disease. In mice with streptozotocin (STZ)-induced type 1 diabetes, meprin α and meprin β double deficiency resulted in a more severe form of kidney injury [12], suggesting that meprins protect against DN. Glomerular meprin expression has also been documented in rats with glomerulonephritis [3] and mice with DN [13]. However, the mechanism(s) by which meprins modulate diabetic kidney injury are not fully understood. Several studies have contributed to knowledge on potential mechanisms via identification of meprin targets present in the kidney [14–29], The levels of urinary meprins and two meprin targets (nidogen-1 and MCP-1) positively correlated with the severity of kidney injury in diabetic African American men [30]. Many of the known meprin targets also play a role in the pathology of DN (e.g. inflammation and fibrosis). However, the metabolic pathways impacted by proteolytic processing of meprin targets are not known. This knowledge is important in understanding how meprins modulate diabetic kidney injury and in development of new diagnostic and therapeutic targets. Metabolomics tools have shown great promise in development of diagnostic and prognostic biomarkers as well as in advancing our understanding of the molecular mechanisms underlying the pathology of CKD [31–38]. In a metabolome-wide association study, kidney function-associated metabolites were shown to have potential as filtration markers which could serve for assessment of kidney function [33]. Experiments with non-obese diabetic mice showed differences in the plasma metabolites of mice which progressed to type 1 diabetes when compared to non-diabetic counterparts [39]. Metabolomics profiling of serum, urine, and renal extracts from rats with DN also identified several DN-related metabolites which included higher levels of allantoin and uric acid [32]. The objective of the current study was to use meprin β knockout mice (in which the meprin β gene is disrupted) and global metabolomics analysis to gain understanding of the mechanisms by which meprin β modulate kidney injury by identifying meprin β-associated changes in plasma and urine metabolites.

## Methods

### Experimental animals

Wild-type (WT) and meprin β knockout (βKO) male mice on a C57BL/6 background were used for these studies. The meprin βKO mice were generated by the laboratory of Dr. Judith S. Bond at Pennsylvania State University College of Medicine in Hershey Pennsylvania and bred to obtain experimental mice which were housed at the Laboratory Animal Resource Unit (LARU) of North Carolina A&T State University. The WT mice were purchased from Charles River Laboratories at age 6 weeks and subsequently housed at LARU. The mice were housed in group cages (up to 5 mice per cage), maintained on a 12:12 h light:dark cycle, and provided rat chow and water ad libitum. The protocols used were reviewed and approved by the North Carolina A&T State University Institutional Animal Care and Use Committee (IACUC). The WT mice express both meprin A (α-α and α-β) and meprin B (β-β) protein isoforms while the meprin β KO mice only express homomeric meprin A (α-α) and are deficient in meprin B (β-β) and the heterodimeric form of meprin A (α-β). The meprin β KO mice have no obvious phenotype under normal physiology but demonstrate key differences under renal pathological conditions. For each genotype, the mice were randomly allocated to two treatment groups, STZ (*n* = 10) or sodium citrate (NaC) (*n* = 6).

### Induction of diabetes

Type 1 diabetes was induced at age 8 weeks by intraperitoneal injection of low dose STZ (50 mg/kg body weight) in 10 mM NaC buffer (pH 4.5) daily for 5 consecutive days following the protocol described by Tesch and Allen (2007) [40], and recommended by the Animal Models of Diabetic Complications Consortium (AMDCC). Control mice were injected with NaC buffer vehicle. Diabetes was confirmed by determining fasting blood glucose levels using a glucose meter at 10 days post-STZ injection. Mice with fasting glucose levels ≥280 mg/dL were considered diabetic. STZ-injected mice with fasting blood glucose levels < 280 mg/dL (*n* = 1 WT and *n* = 2 βKO) were culled and eliminated from the study. The body weights were monitored on a weekly basis.

### Collection of urine and blood

Blood and urine samples were collected at 4 and 8 weeks post-STZ injection. Blood was obtained by tail nicking at week 4 and by cardiac puncture at week 8 when the mice were sacrificed. The blood samples were collected into heparinized tubes, centrifuged to obtain plasma, and stored at − 80 °C until used. Urine samples were obtained by bladder massage into sterile petri dishes and stored at − 80 °C until used. The mice were sacrificed at week 8 by CO_2_ asphyxiations.

### Biochemical assessment for kidney injury

To confirm kidney injury, we performed assays for biomarkers of kidney injury, namely; urinary albumin and creatinine, plasma neutrophil gelatinase-associated lipocalin (NGAL), kidney injury molecule-1 (KIM-1), and cystatin C. The assays for creatinine utilized a colorimetric based enzymatic assay kit (Diazyme Laboratories, Poway CA) while assays for albumin, NGAL, KIM-1, and cystatin C utilized quantitative sandwich enzyme immunoassay kits (Exocell, Philadelphia, PA for albumin and R&D Systems, Minneapolis, MN for the rest). The assays were performed according to the manufacturers’ instructions. The samples were run in duplicate and the optical density (OD) read using a M200 Pro multimode plate reader (Tecan, USA). For NGAL, KIM-1 and cystatin C, the standard curves were plotted using a 4-parameter logistic curve fit (4-PL) using GraphPad Prism 7.0 Software. The ELISA data were analyzed by 2-way ANOVA with multiple comparisons. *P* values ≤0.05 were considered significant. For the fold-change in urinary KIM-1 the levels for non-diabetic NaC treated mice were used as the reference point.

### Sample preparation for metabolomics analysis

Urine samples were thawed, vortexed and centrifuged at 16,000×g for 4 min. The supernatant (40 μL) was transferred to a low protein-binding microcentrifuge tube for each individual, quality control (QC), equilibration (EQ), and total study pool sample. The internal standard working solution (120 μL; 0.0167 mg/mL Tryptophan-d5 in acetonitrile) was added to all samples, and samples were mixed for 2 min at 5000 rpm then centrifuged at 16,000×g for 4 min. Samples were stored at -20 °C until the day of analysis. Samples were vortex mixed for 2 min at 5000 rpm, centrifuged at 16,000×g for 4 min, and the supernatants were transferred to glass autosampler vials for injection on the UPLC-QTOF MS system.

Plasma samples were thawed for 30–60 min on ice, followed by mixing at 5000 rpm for 4 min on a multiple-tube vortex mixer and centrifugation at 16,000×g for 4 min. The plasma supernatants (40 μL) were transferred to a low protein-binding microcentrifuge tube for each individual, QC, EQ, and total study pool sample. The internal standard working solution (320 μL; 0.0125 mg/mL Tryptophan-d5 in methanol) was added to all samples and were vortexed at 5000 rpm for 2 min then centrifuged at 16,000×g for 4 min. Supernatants (290 μL) were transferred to fresh low protein-binding microcentrifuge tubes and capped with rubber stoppers. Tubes were stored at -80 °C for 1 h prior to lyophilization to complete dryness for 18 h. Samples were stored at -80 °C for 5 days. On the day of analysis, samples were reconstituted in 95:5 acetonitrile:water (125 μL), mixed at 5000 rpm for 10 min, centrifuged at 16,000×g for 4 min, and the supernatants were transferred to glass autosampler vials for injection on the UPLC-QTOF MS system.

### Metabolomics quality control

All samples were prepared and analyzed in a randomized order. 11 μL of urine or plasma from all individual study samples were transferred to one, low protein-binding microcentrifuge tube to create the total pool. These total pools were vortex mixed for 30 s and aliquoted to create QC and column EQ samples. Equilibration samples were injected multiple times at the beginning of the analysis to ensure column equilibration. QC samples were evenly distributed throughout the analytical run every 7–10 samples and used to evaluate technical reproducibility of the data and monitor analytical drift. In addition, a volume of the urine (132 μL), plasma (132 μL), and kidney homogenate (132 μL, data not reported here) total pools were mixed to create a total study pool to aid in data alignment and comparison across the three matrices. The total study pool was used to prepare 9 total study pool samples, and 3 total study pool samples were included in each analysis. The mass spectrometer was calibrated at the beginning of each analytical run, and a leucine enkephalin reference was introduced throughout for lock mass correction during data analysis steps. A mixture of standards was injected at the beginning and the end of the run to evaluate system suitability and instrument drift.

### HILIC UPLC-QTOF MS analysis

A hydrophilic interaction liquid chromatography (HILIC) broad spectrum metabolomics method was used to detect metabolites. Samples were analyzed by ultra performance liquid chromatography–quadrupole time-of flight mass spectrometry (UPLC-QTOF MS) using an Acquity I–class connected to a Synapt G2-Si system (Waters, Milford, MA, USA). The UPLC-QTOF MS system was directed by MassLynx 4.1 software to inject and analyze the samples. A volume of 3 μL of each sample was carried by a two solvent system (mobile phase A: 10 mM ammonium acetate in 95:5 acetonitrile:H_2_O with 0.1% Formic Acid and mobile phase B: 10 mM ammonium acetate in 50:50 acetonitrile:H_2_O with 0.1% Formic Acid) with a flow rate of 0.400 mL/min to the Acquity UPLC BEH Amide column (2.1 X 100 mm 1.7 μm). The column temperature was set to 40 °C and the gradient was as follows: t = 0 min, 1% B; t = 1 min, 1% B; t = 12 min, 50% B; t = 14 min, 50% B; t = 15 min, 1% B; t = 19 min, 1% B. A mixture of standards was first injected to check the system suitability, and the column was equilibrated with repeat injections of EQ samples immediately prior to injection of the study samples (individual and QC samples). After separation on the column, samples were directed towards the Synapt G2Si ESI-Q-TOF instrument to be analyzed in positive and negative modes. ESI source settings were as follows: capillary voltage 1.0 kV; sampling cone 40; source offset 80; source temperature 110 °C; desolvation temperature 400 °C; cone gas 50 L/h; desolvation gas 1000 L/h; nebulizer 6.5 bar; lock spray capillary 1.0 kV and lockspray flow rate 3 L/min. Ions were analyzed in MSE continuum mode with trap collision energy ramping from 20 to 30 V for the high energy scan. The scan range was set to 50–1000 m/z with a scanning cycle of 0.6 s in resolution mode (resolution approx. 18,000).

### Metabolomics data analysis

Progenesis QI software (Nonlinear Dynamics, UK) was used to align, pick peaks, deconvolute and annotate compounds. One negative mode sample, a STZ-treated WT 8 week plasma, was removed due to large deviations in total ion intensity. The lock-mass signal was unstable in several samples which created problems with data alignment in the mass dimension and outliers in the multivariate analysis, which led to the exclusion of one sample, a plasma pool, in positive mode and three STZ-treated 4 week plasma samples, two WT and one βKO, in negative mode. The criteria for selecting compounds for further analysis was as follows: retention time greater than 1 min, peak width between 0.1 and 2 min, an abundance greater than 200 in at least 4 of 5 pooled QC samples, and a CV less than 0.4 amongst QC samples when normalized by the total intensity of all compounds picked by the automatic algorithm. The sum of intensities of this compound subset was subsequently used to normalize the intensities of all samples in the matrix before Pareto scaling and multivariate analysis. This is in line with previous approaches for normalization of metabolites in urine samples [41–44]. Unsupervised multivariate analysis (principle components analysis, PCA) was performed using SIMCA 14.1 (Umetrics, Umeå, Sweden) to evaluate data structure and supervised multivariate analysis (orthogonal partial least squares – discriminant analysis, OPLS-DA) was used to determine important group differentiating metabolites. Univariate statistics completed in SAS 9.4 (SAS Institute Inc., Cary, NC, USA). Hypotheses were tested with Exact Wilcoxon Rank Sum or Wilcoxon Rank Sum tests as indicated in Tables 1, 2, 3, 4 and 5. Compounds with a variable importance in projection (VIP) score > 2 in at least one OPLS-DA group comparison were prioritized for identification and interpretation. The HMDB database (www.hmdb.ca) was searched by exact mass in Progenesis QI to annotate compounds. The mass error (< 10 ppm), isotope similarity (> 70%), match between in silico fragmentation results and fragments in high energy spectra (at least one fragment < 12 ppm mass error, not the loss of water) and potential biological relevance were evaluated for passing tolerances before reporting annotated compounds. Compounds referred to as “differentiating” or having “changed levels” throughout the text and included in the tables have VIP ≥ 2 where the jackknifed confidence interval does not include 0 or *p*-value < 0.05.

**Table 1:** PCA/multivariate Analysis models for plasma

	Model	# of Compounds	Total # Samples	# of Components	R2X	R2Y	Q2	# Samples in STZ	# Samples in NaC
Positive	PCA All Samples	2414	62	7	0.947		0.85		
	PCA 4 wks	2414	28	7	0.96		0.841		
	PCA WT STZ v WT NaC 4 wk	2414	14	4	0.937		0.759		
	OPLSDA WT STZ v WT NaC 4 wk	2414	14	1 + 1	0.75	0.619	0.0699	9	5
	PCA βKO STZ v βKO NaC 4wk	2414	14	4	0.93		0.756		
	OPLSDA βKO STZ v βKO NaC 4wk	2414	14	1 + 3	0.908	0.956	0.788	8	6
	PCA 8 wks	2414	28	5	0.942		0.834		
	PCA WT STZ v WT NaC 8 wk	2414	13	5	0.967		0.829		
	OPLSDA WT STZ v WT NaC 8 wk	2414	13	1 + 2	0.845	0.912	0.764	8	5
	PCA βKO STZ v βKO NaC 8wk	2414	14	4	0.962		0.912		
	OPLSDA βKO STZ v βKO NaC 8wk	2414	14	1 + 7	0.984	1	0.731	8	6
Negative	PCA All Samples	633	57	8	0.877		0.711		
	PCA 4 wks	633	25	3	0.76		0.385		
	PCA WT STZ v WT NaC 4 wk	633	12	4	0.925		0.558		
	OPLSDA WT STZ v WT NaC 4 wk	633	12	1 + 2	0.873	0.962	0.876	7	5
	PCA βKO STZ v βKO NaC 4wk	633	13	2	0.58		0.23		
	OPLSDA βKO STZ v βKO NaC 4wk	633	13	1 + 1	0.528	0.958	0.876	7	6
	PCA 8 wk	633	26	3	0.759		0.478		
	PCA WT STZ v WT NaC 8 wk	633	12	3	0.866		0.497		
	OPLSDA WT STZ v WT NaC 8 wk	633	12	1 + 2	0.861	0.973	0.907	7	5
	PCA βKO STZ v βKO NaC 8wk	633	14	3	0.759		0.174		
	OPLSDA βKO STZ v βKO NaC 8wk	633	14	1 + 1	0.498	0.871	0.686	8	6

**Table 2:** PCA/multivariate Analysis models for urine

	Model	# of Compounds	Total # Samples	# of Components	R2X	R2Y	Q2	# Samples in STZ	# Samples in NaC
Positive	PCA All Samples	3513	54	5	0.781		0.663		
	PCA 4 wks	3513	24	2	0.711		0.627		
	PCA WT STZ v WT NaC 4 wk	3513	13	2	0.783		0.676		
	OPLSDA WT STZ v WT NaC 4 wk	3513	13	1 + 1	0.775	0.993	0.981	9	4
	PCA βKO STZ v βKO NaC 4wk	3513	11	2	0.770		0.634		
	OPLSDA βKO STZ v βKO NaC 4wk	3513	11	1 + 0	0.598	0.596	0.444	8	3
	PCA 8 wks	3513	24	4	0.803		0.641		
	PCA WT STZ v WT NaC 8 wk	3513	13	2	0.774		0.648		
	OPLSDA WT STZ v WT NaC 8 wk	3513	13	1 + 1	0.642	0.937	0.916	8	5
	PCA βKO STZ v βKO NaC 8wk	3513	11	3	0.839		0.546		
	OPLSDA βKO STZ v βKO NaC 8wk	3513	11	1 + 4	0.893	0.999	0.841	6	5
Negative	PCA All Samples	4116	53	8	0.753		0.486		
	PCA 4 wks	4116	24	4	0.692		0.477		
	PCA WT STZ v WT NaC 4 wk	4116	13	2	0.632		0.453		
	OPLSDA WT STZ v WT NaC 4 wk	4116	13	1 + 3	0.73	1	0.983	9	4
	PCA βKO STZ v βKO NaC 4wk	4116	11	2	0.566		0.254		
	OPLSDA βKO STZ v βKO NaC 4wk	4116	11	1 + 0	0.333	0.644	0.451	8	3
	PCA 8 wk	4116	24	4	0.646		0.327		
	PCA WT STZ v WT NaC 8 wk	4116	13	4	0.767		0.311		
	OPLSDA WT STZ v WT NaC 8 wk	4116	13	1 + 0	0.409	0.953	0.921	8	5
	PCA βKO STZ v βKO NaC 8wk	4116	11	2	0.574		0.174		
	OPLSDA βKO STZ v βKO NaC 8wk	4116	11	1 + 6	0.894	1	0.816	6	5

**Table 3:** Total numbers of differentiating metabolites in the positive and negative modes at 4 and 8 weeks post-STZ

		4 Weeks post STZ					8 weeks post-STZ				
		Positive Mode		Negative Mode		Total	Positive Mode		Negative Mode		Total
		Unknown	annotated	Unknown	annotated		Unknown	annotated	Unknown	annotated	
Plasma	βKO Only	118	4	71	5	198	69	0	77	1	147
	WT only	47	2	105	5	159	236	9	122	7	374
	Both WT and βKO	52	4	108	7	171	85	5	113	7	210
Urine	βKO Only	7	1	14	2	24	120	9	244	14	387
	WT only	2475	30	2330	39	4874	1761	22	1636	18	3437
	Both WT and βKO	95	13	98	20	226	641	21	674	32	1368

**Table 4:** Summary of differentiating plasma metabolites at 4- and 8-weeks post STZ with a 2 ≤ FC and/or 2 ≤ VIP. A variable importance in projection (VIP) score 2 ≤ are considered to have strong predictive value. Meprin expression associated metabolites (in either WT or meprin βKO mice only)

	4 Weeks post STZ				8 weeks Post STX			
	Metabolite	VIP	*P*-value*	FC**	Metabolite	VIP	*P*-value*	FC**
WT Only	3-Methoxy-4-hydroxyphenylethyleneglycol sulfate	0.7	0.003	6.6	3-Methoxy-4-hydroxyphenylethyleneglycol sulfate	0.5	0.01	9.1
	N-Heptanoylglycine	0.6	0.005	-5.2	3-Aminosalicylic acid	0.9	0.045	3.6
	3-Methoxytyrosine	0.5	0.018	−3.8	LysoPC(16:1(9Z))	3	0.019	−3.6
	7-Methoxy-5-prenyloxycoumarin	0.6	0.042	2	5-Acetyl-2,4-dimethyloxazole	0.4	0.003	3.1
					Guanine	0.3	0.019	2.7
					Taurocholic acid	3.2	0.019	2.5
					9′-Carboxy-gamma-chromanol	0.5	0.019	2.5
					Riboflavin	0.4	0.019	2.5
					N-Acetylleucine	1	0.005	2.2
					Indoxyl sulfate	5.3	0.003	2.1
					2-Phenylglycine	0.3	0.003	2.1
					N-Heptanoylglycine	0.2	0.03	−2.1
					7-Methoxy-5-prenyloxycoumarin	0.4	0.019	2
βKO Only	Porric acid B	0.5	0.021	3.7	Isovalerylalanine	0.7	0.016	−2.5
	3-Methoxy-4-hydroxyphenylethyleneglycol sulfate	1.7	0.047	3.3				
	L-Acetylcarnitine	1	0.021	3.1				
	D-1,5-Anhydrofructose	0.8	0.01	−3.1				
	Hexanoylcarnitine	0.5	0.042	−2.2				
	N-Acetylleucine	1	0.021	2				
	Tyrosyl-Valine	0.3	0.016	2				

*p*-value* : *p*-value for Exact Wilcoxon Rank Sum Test

FC**: A positive or negative fold change indicates median of STZ > median of NaC

**Table 5:** Summary of differentiating plasma metabolites at 4- and 8-weeks post STZ with a 2 ≤ FC and/or 2 ≤ VIP. A variable importance in projection (VIP) score 2 ≤ are considered to have strong predictive value. Diabetes/STZ associated plasma metabolites independent of meprin β expression/deficiency (change in both WT and meprin βKO mice)

4 Weeks post STZ							8 weeks post-STZ						
	WT			βKO				WT			βKO		
	VIP	*P*-value*	FC**	VIP	*P*-value*	FC**		VIP	*P*-value*	FC**	VIP	*P*-value*	FC**
Cortisol	1.7	0.003	23.2	0.9	0.013	2.4	Indole-3-carboxilic acid-O-sulphate	0.5	0.003	6.2	0.1	0.01	935.1
Indole-3-carboxilic acid-O-sulphate	0.7	0.005	4.7	0.2	0.013	10.3	Equol 7-O-glucuronide	0.3	0.002	4.5	0.4	0.02	3.2
LysoPC(16:1(9Z))	5.1	0.002	−4	4.8	0.026	−2.3	Cortisol	0.9	0.003	4.2	0.8	0.026	4.4
Fenoprofen glucuronide	0.7	0.005	3.4	1.4	0.013	3.9							
L-Carnitine	0.9	0.019	−3.3	1.2	0.01	−3.2	Fenoprofen glucuronide	0.7	0.003	3.6	0.8	0.01	3.1
APGPR Enterostatin	1.1	0.005	−3	1.5	0.013	−4	Tyrosyl-Valine	0.3	0.019	3.4	0.3	0.033	1.9
Hippuric acid	0.9	0.048	2.4	1.6	0.013	2.7	3-Methoxy-4-hydroxyphenylethyleneglycol sulfate	0.7	0.03	3.2	0.9	0.042	2.7
3,4-Dihydroxyphenylglycol O-sulfate	0.5	0.005	2.4	0.4	0.036	1.2	Hippuric acid	1.2	0.003	3.2	1.2	0.012	2.5
Isovalerylalanine	0.5	0.005	−2.3	0.8	0.013	−2.2							

*p*-value* : *p*-value for Exact Wilcoxon Rank Sum Test

FC**: A positive or negative fold change indicates median of STZ > median of NaC

All raw and processed analytical data and associated metadata have been deposited into the NIH Common Fund’s Data Repository and Coordinating Center (supported by NIH grant, U01-DK097430) website, http://www/metabolomicsworkbench.org, where it has been assigned Metabolomics Workbench Project IDs PR000409 (plasma) and PR000410 (urine). The data is directly accessible at the following websites: http://www.metabolomicsworkbench.org/data/DRCCMetadata.php?Mode=Project&ProjectID=PR000409OR https://bit.ly/2C95oe1

http://www.metabolomicsworkbench.org/data/DRCCMetadata.php?Mode=Project&ProjectID=PR000410OR https://bit.ly/2tUReZK

## Results

### Meprin β expression is associated with higher levels of kidney injury

**Fig. 1:**
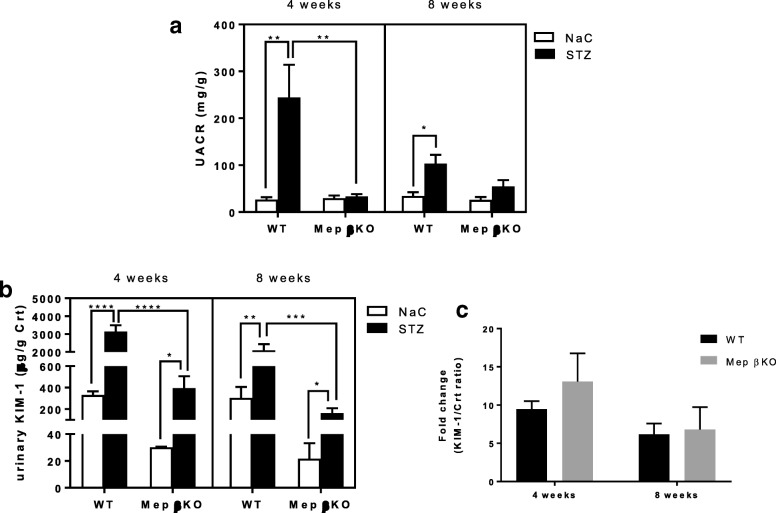
Assessment of Kidney injury. Panel **a** Urinary albumin to creatinine ratio (UACR) for WT and Meprin βKO mice, Panel **b** Urinary KIM-1 levels, and Panel **c** fold change in KIM-1. Spot urine samples were collected by bladder massage at 4 and 8 weeks post-STZ injection. ELISA kits (R&D Systems) were used for albumin and KIM-1 assays and calorimetric assays kits (Diazyme Inc) for creatinine assays. Urinary KIM-1 concentrations were normalized to urinary creatinine concentrations. The fold change in KIM-1/Creatinine ratio (panel B) were calculated relative to the concentrations in the control mice for each genotype. Data are represented as mean ± SEM. *, *p* < 0.05; **, *p* < 0.01; ***, *p* < 0.001; ****, *p* < 0.0001

**Fig. 2:**
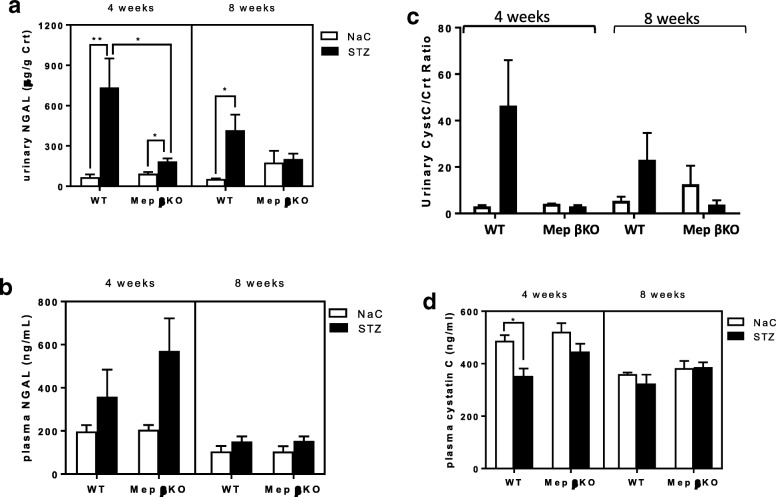
Urinary and plasma NGAL and cystatin C levels for WT and meprin βKO mice. Blood and urine samples were collected at 4 and 8 weeks post-STZ injection for analysis. NGAL and cystatin concentrations were normalized to urinary creatinine concentrations. Data are represented as mean ± SEM. * p < 0.05; ** p < 0.01

Overall, WT mice with STZ-induced type 1 diabetes had higher levels of kidney injury when compared to meprin βKO mice. The urinary albumin to creatinine ratios (UACR) were significantly higher for WT mice at both 4 (*p* = 0.0042) and 8 weeks (*p* = 0.0383) post-STZ injection. In contrast, meprin βKO mice had a modest increase in UACR at 8 weeks post-STZ (Fig. 1, panel A). The ratio of urinary KIM-1 to creatinine showed significant increases in both WT and meprin βKO mice at 4 and 8 weeks post-STZ but the baseline levels were higher in WT mice when compared to meprin βKO counterparts (Fig. 1, panel B). There were no significant differences between the two genotypes in the fold-change of the urinary KIM-1/Creatinine ratios at either 4 or 8 weeks (Fig. 1, panel C). The ratios of urinary NGAL to creatinine were also significantly higher at 4 weeks in diabetic WT mice when compared to diabetic meprin βKO counterparts (Fig. 2, panel A). In contrast, there were no significant changes in plasma NGAL for either genotype at 4 or 8 weeks post-STZ injection (Fig. 2, panel B). While plasma cystatin C levels decreased in WT mice at 4 weeks post-STZ, the decrease was not sustained at 8 weeks post STZ and was not observed in the meprin βKO mice (Fig. 2, panel D). Furthermore, there were no significant differences in the urinary cystatin C levels (Fig. 2, panel C) even though there was a modest increase in WT mice which also showed great individual variations.

### Meprin expression alters plasma and urinary metabolite profiles in mice with STZ-induced type 1 diabetes

**Fig. 3:**
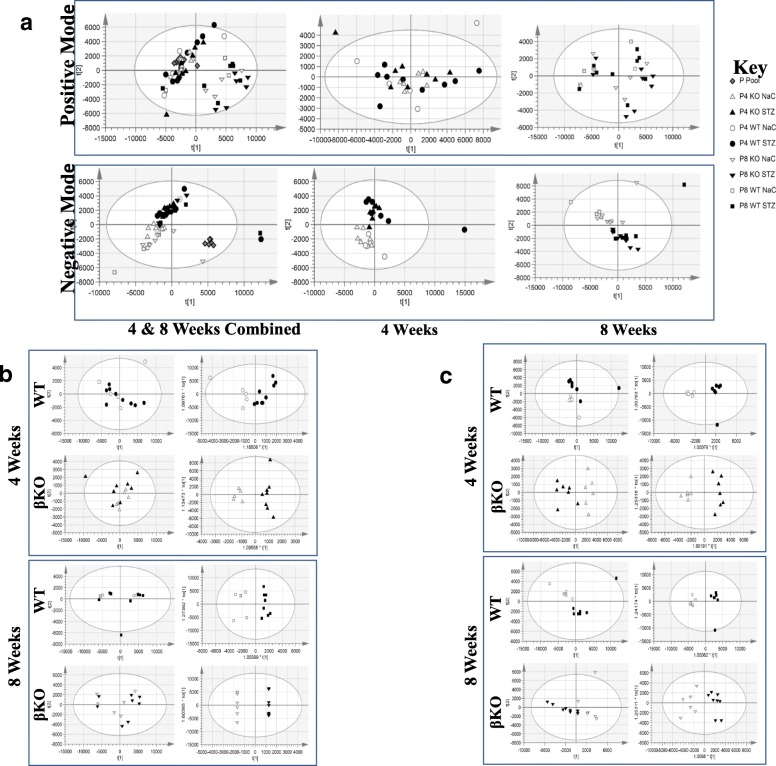
Scores plots for multivariate statistical analyses of metabolites in plasma. Blood samples were collected at 4-week post STZ injection by tail nick and 8-week by cardiac puncture and processed to obtain plasma. Panel **a**: Unsupervised analysis principal components analysis (PCA) demonstrating overall structure and variability within the large LC-MS dataset for all, 4 week only and 8 week only plasma samples in either positive or negative electrospray ionization modes. Panel **b**: Side-by-side scores plots for the unsupervised PCA and supervised OPLS-DA multivariate analyses for each genotype in positive mode. Panel **c**: Side-by-side scores plots for the unsupervised PCA and supervised OPLS-DA multivariate analyses for each genotype in negative mode. These plots visualize (along with Tables 1, 2 and 3 inputs and fit statistics to aid interpretation) the STZ treatment effect in the data subset PCA and the effectiveness of OPLS-DA to uncover metabolite variables (loadings) capable of discriminating the two treatment groups

**Fig. 4:**
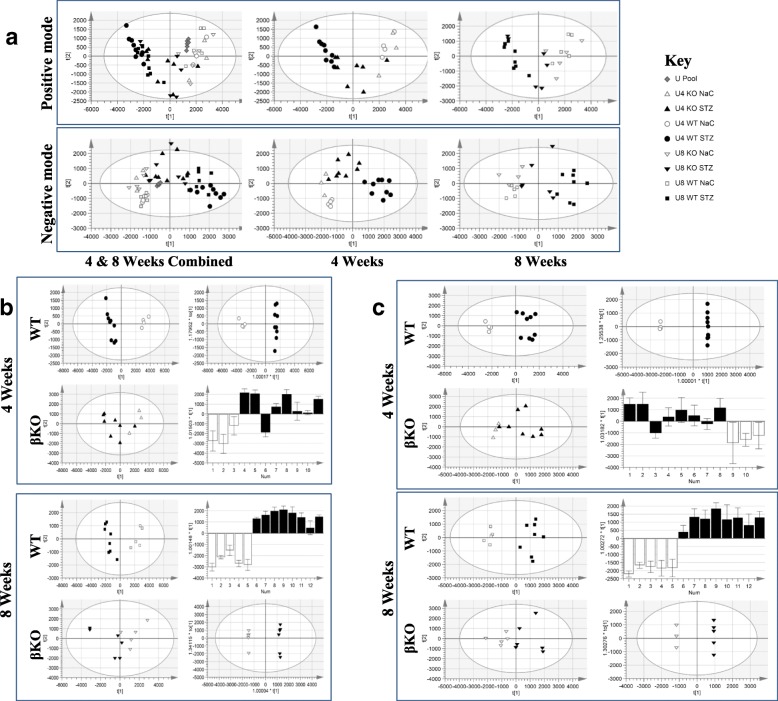
Scores plots for multivariate statistical analyses of metabolites in urine. Spot urine samples were collected at 4- and 8-week post STZ injection. Panel **a**: Unsupervised principal components analysis (PCA) demonstrating overall structure and variability within the large LC-MS dataset for all, 4 week only, and 8 week only urine samples in either positive or negative electrospray ionization modes. Panel **b**: Side-by-side scores plots for the unsupervised PCA and supervised OPLS-DA multivariate analyses for each genotype in positive mode. Panel **c**: Side-by-side scores plots for the unsupervised PCA and supervised OPLS-DA multivariate analyses for each genotype in negative mode. These plots visualize (along with Tables 1, 2 and 3 inputs and fit statistics to aid interpretation) the STZ treatment effect in the data subset PCA and the effectiveness of OPLS-DA to uncover metabolite variables (loadings) capable of discriminating the two treatment groups

Our data show distinct changes in the profiles of metabolites present in plasma and urine associated with STZ-induced type 1 diabetes and diabetic kidney injury in WT and meprin βKO mice. The PCA score plot for all plasma samples in positive mode showed duration of diabetes status was the major source of variability (Fig. 3 panel A; R^2^X(cum) = 0.947; Q^2^(cum) = 0.85). In contrast, the overall PCA of urine and plasma negative mode datasets indicated that the strongest source of variability was due to diabetes status (Fig. 3 panel A and Fig. 4 panel A; Model fit statistics (Tables 1 and 2). QC samples were tightly clustered, and near the center of the datasets, demonstrating technical reproducibility of the data. When compared to plasma, the number of differentiating metabolites in urine were much higher for both genotypes reflecting the control over homeostasis in plasma (Table 3 and Additional file 1: Tables S1, S2, S3,S4, S5, S6, S7 and S8). Similarly, separation by treatment type was more pronounced in urine in the PCA for most models. In addition to meprin-associated changes in metabolite levels (Tables 4 and 6), several metabolites differentiated diabetic from non-diabetic controls in both WT and meprin βKO mice (Tables 5 and 7), suggesting an association with diabetic status but independent on meprin expression/activity or deficiency. For both genotypes, there were more differentiating metabolites at 8 weeks post-STZ when compared to 4 weeks post-STZ, suggesting an association with severity of complications of diabetes (Table 3).

**Table 6:** Summary of differentiating urinary metabolites in WT and meprin βKO mice at 4- and 8-weeks post STZ with a 2 ≤ FC and/or 2 ≤ VIP. A variable importance in projection (VIP) score 2 ≤ are considered to have strong predictive value. Differentiating urinary metabolites that associate with meprin expression (Change in either WT or βKO only)

	4 Weeks post STZ				8 Weeks post STZ			
		VIP	*P*-value*	FC**		VIP	*P*-value*	FC**
WT Only	Cortisol	10.5	0.003	54.9	Cortisol	8.2	0.002	21.6
	9,13-Dihydroxy-4-megastigmen-3-one 9-glucoside	1.6	0.003	−22.9	Homovanillic acid sulfate	7.8	0.002	21
	Aromadendrin 3,7-diglucoside	1.3	0.02	6.9	Aromadendrin 3,7-diglucoside	2	0.002	13.9
	Hydroxybutyrylcarnitine	1	0.003	−6.2	Pyrogallol-2-O-glucuronide	1.5	0.006	−3.5
	Homovanillic acid sulfate	8.3	0.02	4.1	Phloretin 2′-O-glucuronide	1.3	0.002	3.4
	Taurocholic acid	0.5	0.003	4	Prenyl glucoside	2	0.002	−3.1
	N-Heptanoylglycine	4.7	0.011	−3.9	Caffeic acid 3-sulfate	4.7	0.002	2.3
	Cicerin 7-(6-malonylglucoside)	3.5	0.003	3.7	4-Hydroxyphenylpyruvic acid	1.7	0.003	2.3
	Porric acid B	3.3	0.003	−3.7	Tyrosyl-Valine	1.4	0.006	2.3
	Phloretin 2′-O-glucuronide	1.2	0.003	3.6	Dibutyl malate	1.7	0.002	2.1
	Phenylacetylglycine	0.4	0.003	−3.4	Ergothioneine	0.3	0.002	2.1
	20-oxo-leukotriene B4	2.1	0.003	3.3	Taurocholic acid	0.3	0.002	2
	Tyrosyl-Valine	1.8	0.003	3.3	Indole-3-carboxilic acid-O-sulphate	4.9	0.002	1.9
	LysoPC(16:1(9Z))	0.5	0.003	2.9	7-Methoxy-5-prenyloxycoumarin	2.4	0.002	1.9
	3b,8b-Dihydroxy-6b-angeloyloxy-7(11)-eremophilen-12,8-olide	1.8	0.003	2.7	5-Acetyl-2,4-dimethyloxazole	2.2	0.002	1.7
	Indole-3-carboxilic acid-O-sulphate	1.7	0.003	2.7	3,5-Dihydroxy-3′,4′-dimethoxy-6,7-methylenedioxyflavone 3-glucuronide	5.6	0.004	−3.7
	Caffeic acid 4-sulfate	2.5	0.003	−2.7	3-Formyl-6-hydroxyindole	1.2	0.017	3.6
	Phenylpropionylglycine	4.8	0.011	−2.4	Formononetin 7-O-glucuronide	2.3	0.004	1.7
	Ergothioneine	0.3	0.003	2.1	Indoxyl glucuronide*	3.2	0.004	−1.7
	Hippuric acid	1.4	0.034	−2				
βKO Only	4-Methoxyphenylacetic acid	2.3		1.4				
	3-Methoxy-4-hydroxyphenylethyleneglycol sulfate	14.2		1.3				
	Riboflavin	2.5		−1.3				

*p*-value* : *p*-value for Exact Wilcoxon Rank Sum Test

FC**: A positive or negative fold change indicates median of STZ > median of NaC

**Table 7:** Summary of differentiating urinary metabolites in WT and meprin βKO mice at 4- and 8-weeks post STZ with a 2 ≤ FC and/or 2 ≤ VIP. A variable importance in projection (VIP) score 2 ≤ are considered to have strong predictive value. Differentiating Urinary metabolites that are independent of meprin β expression (levels change in both WT and meprin βKO mice)

4 Weeks post STZ							8 weeks post-STZ						
	WT			βKO				WT			βKO		
	VIP	*P*-value*	FC**	VIP	*P*-value*	FC**		VIP	*P*-value*	FC**	VIP	*P*-value*	FC**
a-L-threo-4-Hex-4-enopyranuronosyl-D-galacturonic acid	2.8	0.003	− 172.7	3.9		−20.8	a-L-threo-4-Hex-4-enopyranuronosyl-D-galacturonic acid	2.4	0.003	−22.5	3.3	0.052	−4.3
S-(Formylmethyl)glutathione	1.4	0.003	−31	2.7		−5.6	9,13-Dihydroxy-4-megastigmen-3-one 9-glucoside	1.7	0.002	−19.1	1.5	0.004	−3.5
1-Pyrroline-4-hydroxy-2-carboxylate	2.5	0.003	−13.2	2.6		−3.6	S-(Formylmethyl)glutathione	1.2	0.002	−8.4	2.6	0.126	−2.3
Avenic acid B	1.4	0.003	−7.3	2.5		−4.8	1-Pyrroline-4-hydroxy-2-carboxylate	2.6	0.002	−7.7	2.1	0.017	−2.8
Glycerophosphocholine	3.9	0.003	−6.9	2.6		−3	Hydroxybutyrylcarnitine	1.1	0.002	−6.5	1.7	0.004	−5.4
Nicotinic acid	3.3	0.003	−5.9	3.8		−3.1	Cicerin 7-(6-malonylglucoside)	4	0.006	5.8	1.4	0.004	1.8
Indole-3-carboxylic acid	2.4	0.003	−4.8	2.3		−2.2	Avenic acid B	1.5	0.002	−5.8	1.5	0.009	−1.7
Pyrogallol-2-O-glucuronide	1.7	0.003	−4.8	2.2		−2.6	Glycerophosphocholine	4	0.002	−4.8	4.1	0.017	−2.2
N-a-Acetyl-L-arginine	2.9	0.003	−3.3	2.4		−1.6	Indole-3-carboxylic acid	2.7	0.002	−4.8	2.5	0.004	−3
N-Carboxyethyl-g-aminobutyric acid	1.5	0.003	−3.3	2.2		−2.8	N-Carboxyethyl-g-aminobutyric acid	2	0.002	−4.8	1.8	0.03	−1.8
Galactonic acid	4.3	0.003	−3.1	5.2		−2.5	Nicotinic acid	3.3	0.002	−3.6	3.9	0.004	−2.4
4-Hydroxyphenylpyruvic acid	2.5	0.003	2.6	2.2		4.8	Porric acid B	3.5	0.002	−3.4	3.3	0.052	−1.6
Symmetric dimethylarginine	3.5	0.003	−2.6	4.3		−2.3	1,3,5-Trimethoxybenzene	2.1	0.002	−3.1	1.5	0.009	−1.6
7-Methoxy-5-prenyloxycoumarin	2.6	0.003	2.5	2.3		1.6	Phenylacetylglycine	0.4	0.002	−3	0.4	0.004	−2.6
Indoxylglucuronide	1.8	0.034	−2.5	3.5		−2	N-Acetylleucine	6.4	0.006	2.7	5.2	0.329	1.3
Caffeic acid 3-sulfate	6.6	0.003	2.4	5.8		4.2	20-oxo-leukotriene B4	1.7	0.003	2.6	1.8	0.009	2.3
Genistein 5-O-glucuronide	3.2	0.003	2.3	2.9		1.2	N-Heptanoylglycine	4.4	0.006	−2.6	6.7	0.017	−3.9
N-Acetylleucine	7.5	0.02	2.2	7		2	Indole-3-carboxilic acid-O-sulphate	1.8	0.002	2.4	0.6	0.004	3.3
Betaine	4.1	0.006	2	3.2		1.5	L-Carnitine	4.6	0.002	−2.4	6.2	0.03	−1.7
Indole-3-carboxilic acid-O-sulphate	3.9	0.02	1.6	2.4		1.8	D-1,5-Anhydrofructose	0.5	0.003	−2.2	1.1	0.004	−2.3
5-Acetyl-2,4-dimethyloxazole	2.3	0.003	1.7	2.6		1.7	Galactonic acid	3.8	0.003	−2.1	4.2	0.177	−2.1
Lotaustralin	2.5	0.003	1.8	2.4		1.6	Genistein 5-O-glucuronide	2.6	0.03	1.8	3.3	0.004	1.6
Fenoprofen glucuronide	6.7	0.003	1.6	10		1.5	Equol 7-O-glucuronide	3.5	0.003	1.7	4.6	0.004	1.5
Equol 7-O-glucuronide	3.2	0.003	1.6	4.1		1.5	Isovalerylalanine	4.7	0.03	−1.5	7.6	0.052	−3.7
							Indolelactic acid	4.6	0.065	−1.5	5.8	0.082	−1.5

*p*-value* : *p*-value for Exact Wilcoxon Rank Sum Test

FC**: A positive or negative fold change indicates median of STZ > median of NaC

#### Meprin expression-associated differentiating metabolites in plasma

Metabolites with changed levels in either WT or meprin βKO mice only were considered to be associated with meprin β expression/activity or meprin β deficiency. While plasma PCA and OPLS-DA models or diabetes status were overall of good quality (Table 1. Plasma model statistics; Fig. 3), PCA models showed the source of variability on components 1 and 2 in the positive mode datasets was not treatment group. At 4 weeks post-STZ injection there were significant changes in the levels of 159 plasma metabolites in WT mice only (7 annotated, 152 of unknown identity) (Fig. 3 panel B). Although seven of the annotated plasma metabolites (Table 4) had a 1.5 ≤ FC, they all had VIP ≤ 1 scores, suggesting that they are not strong predictors. The number of differentiating metabolites in the plasma of WT mice at 8 weeks was much higher (16 annotated, 358 unknown). Fifteen of the annotated metabolites had 1.5 ≤ FC but only three had a 2 ≤ VIP score, making them strong predictors. These three are; LysoPC(16:1(9Z)) (FC = -3.6; VIP = 3), taurocholic acid (FC = 2.5,; VIP = 3.2), and indoxyl sulfate (FC = 2.1; VIP = 5.3). Of the 198 differentiating metabolites in the plasma of meprin βKO at 4 weeks, only 9 were annotated, and at 8 weeks, only one out of 147 metabolites was annotated. Furthermore, none of the identified metabolites had a 2 ≤ VIP score.

#### Differentiating plasma metabolites independent of meprin expression

The levels of several metabolites were significantly changed in the plasma of both WT and meprin βKO mice indicating that the changes are associated with the diabetic status but independent of meprin expression/activity or deficiency (Table 5). Of the annotated metabolites at four weeks post-STZ, only one metabolite had 1.5 ≤ FC and a 2 ≤ VIP score in both genotypes, namely; LysoPC(16:1(9Z)) (FC = 4.8; VIP = 5.1). Of interest though, hippuric acid showed significant increases in both genotypes at 4 and 8 weeks post-STZ with fairly strong VIP scores at 8 weeks. Several other metabolites had significant FC in both genotypes but their VIPs were low. For example, at 4 weeks, indole-3-carboxilic acid-O-sulphate had a FC = 4.7 in WT and FC = 10.43 in βKO and at 8 weeks it had a FC = 6.2 in WT and FC = 935.1 in βKO.

### Meprin expression-associated differentiating metabolites in urine

There were distinct changes in metabolite profiles in urine samples from diabetic mice when compared to non-diabetic NaC treated control mice of the same genotype (Tables 3, 6, and 7). Of the annotated metabolites in urine samples from WT mice at 4 weeks, 27 had 1.5 ≤ FC with 10 having 2.0 ≤ VIP scores. At 8 weeks, fifteen metabolites had 1.5 ≤ FC with nine having 2 ≤ VIP scores. Twenty five of the metabolites had significant changes in levels at both 4 and 8 weeks (Table 7) post-STZ suggesting that they are reliably associated with DN and perhaps other complications of diabetes. However, only three of these had 2 ≤ VIP scores at both time points, namely; cortisol, homovanillic acid sulfate, phloretin 2′-O-glucuronide and are thus considered strong predictors for early onset of complications of diabetes such as DN. Interestingly the three identified metabolites that significantly changed levels in the urine of βKO mice at 4 weeks all had a FC ≤ 1.5 even though all their VIP scores were greater than 2 and their levels were not significantly changed at 8 weeks (Additional file 1: Tables S1, S2, S3,S4, S5, S6, S7 and S8). At 8 weeks, there were significant decreases in the levels of three metabolites (which also had strong VIP scores) in urine from βKO mice; 3,5-Dihydroxy-3′,4′-dimethoxy-6,7-methylenedioxyflavone 3-glucuronide (FC = -3.7; VIP = 5.6), pantothenic acid (FC = -1.7, VIP = 4.1), and indoxyl glucuronide (FC = -1.7; VIP = 3.2).

#### Differentiating urine metabolites independent of meprin expression

There were distinct changes in the profiles of several metabolites in urine for both WT and meprin βKO mice at both 4 weeks (33 annotated, 193 unknown) and 8 weeks post-STZ injection (53 annotated, 1315 unknown) (Tables 3 and 7, and Addiitonal file 1: Tables S5-S8). Nine metabolites had 1.5 ≤ FC and 2 ≤ VIP score at both 4 and 8 weeks post-STZ suggesting a strong association with diabetes and diabetes associated-complications such as DN but independent of meprin β expression/activity or deficiency. The nine are; a-L-threo-4-Hex-4-enopyranuronosyl-D-galacturonic acid, 1-Pyrroline-4-hydroxy-2-carboxylate, Glycerophosphocholine, Nicotinic acid, Indole-3-carboxylic acid, Galactonic acid, Genistein 5-O-glucuronide, N-Acetylleucine, and Equol 7-O-glucuronide.

### Meprin expression-associated metabolites that changed in both plasma and urine

Only 3 metabolites had changed profiles in both plasma and urine. The levels of N-heptanoylglycine decreased mice at 4 weeks post-STZ in biofluids from WT mice (FC = − 5.2 in plasma vs FC = -3.9 in urine). However, the VIP scores in plasma were low (VIP = 0.6 vs VIP = 4.7 in urine). In contrast, the levels of taurocholic acid in WT mice increased at 8 weeks post-STZ with comparable FC values (2.5 in plasma 2.0 in urine) and a stronger VIP score in plasma (3.2 in plasma vs 0.3 in urine). For meprin deficient mice, the levels of 3-Methoxy-4-hydroxyphenylethyleneglycol sulfate increased at 4 weeks post-STZ (FC = 3.3, VIP = 1.7 in plasma; FC = 1.3, VIP =14.2 in urine). TCA has previously been shown to discriminate polycystic rat kidneys from healthy kidneys [45]. Another interesting observation was the fact that changes in the levels of the differentiating urine metabolites occurred earlier in diabetic WT mice when compared to meprin βKO counterparts. There were 22 annotated metabolites whose levels changed in the urine of WT mice at 4-week and 8-week post STZ but these changes only showed in meprin βKO mice at 8-week post STZ. (Additional file 1: Tables S1, S2, S3,S4, S5, S6, S7 and S8). This suggests that meprin β deficiency results in a late onset of diabetes-associated complications.

### Metabolic pathways impacted by meprin expression

The annotated metabolites with significantly changed profiles are associated with several metabolic pathways (Table 8). These include; amino acid metabolism, protein degradation, lipid metabolism, nucleotide metabolism, vitamin metabolism, hormone/neurotransmitter, and small molecule metabolites typically dietary sources or gastrointestinal tract/gut microbiota.

**Table 8:** Summary of metabolic pathways for metabolites with significant changes in levels that associate with meprin β expression or deficiency and STZ-induced type 1 diabetes

Metabolite	Increase/decrease	Pathways
Group 1: changed levels in plasma associate with meprin expression or deficiency		
Taurocholic acid	Increases in WT	Bile acids/lipid metabolism
LysoPC(16:1(9Z))	Decreases in WT	Lipid metabolism
Indoxyl sulfate	Increases in WT	oxidative stress
Group 2: 8 changes in urine associate with meprin β expression or deficiency		
Taurocholic acid^a^	Increase in WT	Bile acids (lipid metabolism)
Ergothioneine^a^	Increase in WT	Gut/dietary source (histidine metabolism)
Tyrosyl-Valine^a^	Increase in WT	Protein degradation (aromatic amino acid metabolism)
Prenyl glucoside	Decrease in WT	Gut/dietary (fatty acyl glucoside)
Homovanillic acid sulfate	increase in WT	Catecholamine metabolism
Cortisol	Increase in WT	Steroid hormone synthesis/stress response
Aromadendrin 3,7-diglucoside	Increase in WT	Gut/dietary (flavonoid)
Phloretin 2′-O-glucuronide^a^	Increase in WT	Gut/dietary (flavonoid)
3,5-Dihydroxy-3′,4′-dimethoxy-6,7- methylenedioxyflavone 3-glucuronide	Decrease in βKO	Gut/dietary
Pantothenic acid	Decrease in βKO	Gut/dietary
Indoxyl glucuronide	Decrease in βKO	Indole metabolism/gut microbiota
Group 3: changed levels in plasma associate with STZ-induced type 1 diabetes but independent of meprin β expression		
Hippuric acid^a^	Increase at 4 and 8 weeks	Amino acid metabolism (phenylalanine polyphenol metabolism; uremia)
Indole-3-carboxilic acid-O-sulphate^a^	Increase	Indole metabolism
Fenoprofen glucuronide^a^	Increase	NSAID metabolite
Group 4: Changed levels in urine associate with STZ-induced type 1 diabetes but independent of meprin β expression		
Glycerophosphocholine	Decrease	Lipid metabolism (choline metabolism; glycerophosphocholine)
Nicotinic acid	Decrease	Co-factor and vitamin (nicotinate and nicotinamide metabolism)
1-Pyrroline-4-hydroxy-2-carboxylate	Decrease	Amino acid metabolism (arginine and proline metabolism)
Indole-3-carboxylic acid	Decrease	Gut/Dietary (indole metabolism)
a-L-threo-4-Hex-4-enopyranuronosyl-D-galacturonic acid	Decrease	Carbohydrate metabolism (glucuronic acid derivative)

^a^VIP ≤ 2 but consistent significant increases at both 4 and 8 weeks post STZ

## Discussion

Meprin metalloproteases have been shown to play a role in the pathology of diabetic nephropathy (DN) in humans and in mouse models of DN [8, 9, 12]. However, knowledge of underlying mechanisms is limited. Known meprin β substrates includes several ECM proteins (e.g., collagen II, Collagen IV, laminin, fibronectin, and nodogen-1) [46] and inflammatory mediators (e.g., IL-6, pro-IL18, MCP-1, and thymosin β4/Ac-SDKP) [28]. Imbalances in ECM metabolism and inflammation both contribute to the fibrosis observed in DN. Other meprin targets e.g. protein kinase C (PKC) [21] and the catalytic subunit of protein kinase A (PKA C) [13, 22, 23] modulate signaling pathways involved in inflammation and ECM metabolism. However, it’s not known how proteolytic processing of meprin targets impacts the metabolite environment in the kidneys and other sites where meprins are expressed. The current study employed global metabolomics analysis to evaluate the metabolite profiles of urine and plasma samples from WT mice (which express normal levels of both meprin A and meprin B) and meprin β KO mice which are deficient in meprin B (β-β) and heterodimeric meprin A (α-β) following induction of type 1 diabetes. Blood and urine samples were obtained at 4 and 8 weeks post-STZ which would correspond to early onset of kidney injury. Biochemical assessment of kidney injury using UACR and recently developed proteomic markers (NGAL, KIM-1, and cystatin C) confirmed kidney injury as early as four weeks post-STZ injection. Of the proteomic biomarkers assayed, urinary NGAL and urinary KIM-1 positively correlated with kidney injury and are thus reliable predictors of early diabetic kidney injury. An interesting finding was the high baseline levels of KIM-1 in the non-diabetic WT mice, suggesting that meprins β could predispose kidneys to tubular injury. The data further suggest that deficiency of meprin β can alleviate the progression of DN. A previous study with meprin αβ double knockout mice showed that deficiency of both meprin α and meprin β resulted in more severe kidney injury [47]. While these findings appear to be contradictory, they point to the isoform-specific impact of meprins. Several studies have demonstrated that inflammation is an underlying mechanism in the progression of diabetic kidney injury. Meprin A has anti-inflammatory activities [28, 48] while meprin B plays both pro-inflammatory [24, 27] and anti-inflammatory [26] roles. It has further been shown that the balance of meprin A and meprin B influences the progression of inflammatory bowel disease in experimental mice [49]. It is thus likely that deficiency in meprin B and heterodimeric meprin A (α-β) tilts the balance toward anti-inflammation activities driven by homomeric meprin A (α-α) and thus protects from injury in diabetic kidney disease. This is supported by the finding that meprin A plays a role in the release of the anti-inflammatory peptide ac-SDKP from thymosine β4 [28].

The findings of the current global metabolomics analysis of plasma and spot urine samples from diabetic WT and meprin βKO mice offer insights into the metabolic alterations occurring in diabetes and the role that meprin β plays in modulating diabetic kidney injury and other complications of diabetes. An important finding from the current study is diabetes-associated changes in the levels of 5 plasma metabolites and 25 urinary metabolites unique to WT mice, suggesting an association with meprin β expression/activity. Many of these metabolites have also been previously reported in association with diabetes, which supports a role of meprin β in modulating the progression of complications of diabetes such as DN. Furthermore, the data suggest that meprin β impacts diverse pathways, which include lipid metabolism, amino acid metabolism, nucleotide metabolism, and vitamin metabolism. Diabetes-associated changes in the levels of taurocholic acid, a bile acid, were only significant in WT mice. Furthermore, taurocholic acid was the only metabolite with changed levels in both urine and plasma at both 4 and 8 weeks post-STZ injection. Multiple studies showed that specific bile acids like taurocholic acid stimulate bicarbonate secretion and bile flow [50]. In another study, patients with type 2 diabetes showed higher plasma taurocholic acid levels when compared to healthy subjects [51]. Taurocholic acid has also been shown to discriminate polycystic rat kidneys from healthy kidneys [45], suggesting a link to renal fibrosis. Given the function of bile acid in promoting lipid metabolism, the resistance of meprin βKO mice to taurocholic acid alteration could contribute to other differences in lipid profiles. This resistance is likely linked to meprin expression in the small intestines rather than in kidney tissue. Among the metabolites associated with amino acid metabolism are kynurenic acid, indoxyl sulfate, and hippuric acid. Kynurenic acid is a metabolite of tryptophan and it has been identified as a kidney function marker in several studies [52, 53]. In the current study, urinary kynurenic acid levels decreased in diabetic WT mice at both 4 and 8 weeks, but not in diabetic meprin βKO mice. This change indicates compromised kidney function in amino acid metabolism, but also suggests meprin β may mediate the metabolism of tryptophan. Furthermore, the levels of indoxyl sulfate increased in urine from WT mice at both 4- and 8-week post STZ, while its levels increased in urine from meprin βKO mice at 8-week but not 4-week post STZ. In plasma, the increased levels of indoxyl sulfate were found only in WT mice at 8-week post STZ. Indoxyl sulfate is also a metabolite of tryptophan, considered a microbiota-derived uremic solute and related with chronic kidney disease [54]. In patients with chronic kidney disease, the serum and urine levels of indoxyl sulfate increased markedly and were shown to promote the progression of renal failure [55, 56]. The differences in indoxyl sulfate level changes in the current study indicate the late onset of kidney injury in meprin βKO mice when compared to WT mice. The levels of hippuric acid increased in the urine of WT mice at both 4- and 8-week. Hippuric acid is involved in phenylalanine metabolism, considered a microbiota-derived uremic solute and related to chronic kidney disease [54]. Both hippuric acid and its precursor benzoic acid have been implicated in early-stage DN [43]. Plasma levels of hippuric acid were shown to be elevated in haemodialysed patients with chronic renal failure when compared to healthy controls and hospital patients without kidney disease [57]. Several differentiating metabolites are involved in vitamin metabolism. We did not observe changes in urinary excretion of riboflavin in WT mice, but the urinary excretion of riboflavin in meprin βKO mice decreased at both 4 weeks and 8 weeks. Meanwhile, riboflavin level in the plasma of WT mice increased at 8 weeks post-STZ injection. Increased urinary excretion of riboflavin was previously observed in rats with STZ-induced diabetes [58]. Furthermore, dietary riboflavin was shown to have protective effects on DN in STZ-induced diabetic rats [59]. Thus, if the decreased urinary riboflavin is caused by the body conservation of riboflavin, it may protect meprin β null mice against diabetic kidney injury. Changes in the levels of N1-methyl-pyridone-3-carboxamide, indicate the involvement of meprin β on vitamin B6 catabolism, nicotinamide-adenine dinucleotide degradation, and biotin metabolism in response to diabetic conditions [60]. The changes of multiple vitamin metabolites suggest system wide mediation of meprin β. This could be via impacting of microbial populations in the small intestines where meprins are abundantly expressed. Meprin β in the small intestine is required for detachment of mucin, which is important for protecting the host epithelium from bacteria [61]. Such protective function of meprin β also corroborates the different microbial populations between WT and meprin βKO mice. Another interesting finding in the current study is meprin expression associated changes in the profiles of several hormone or neurotransmitter metabolites, such as cortisol, and homovanillic acid sulfate. Previous studies demonstrated that serum cortisol levels were significantly related to retinopathy and neuropathy, but not nephropathy [62]. The fold change in urinary cortisol levels was significant in WT mice at both 4- and 8-weeks post-STZ (54.9 and 21.6), while no such alteration was seen in meprin β null mice. Furthermore, the fold change in plasma cortisol was more significant in WT than meprin β null mice at 4 weeks post-STZ (23.2 vs 2.4). 3-Methoxy-4-hydroxyphenylethyleneglycol sulfate is a major metabolite of noradrenaline. Its formation in brain is used as an estimate of the norepinephrine formation rate [63]. It was also identified as a potential marker for uremia. It significantly increased in subjects with end stage renal disease when compared with healthy controls [64]. Homovanillic acid sulfate is a major catecholamine metabolite while urinary homovanillic acid sulfate was shown to be a predictive early biomarker of acute kidney injury after pediatric cardiac surgery [65]. Interestingly, only two meprin-expression (N-Heptanoylglycine and Taurocholic acid) and one meprin-deficiency associated metabolites (3-Methoxy-4-hydroxyphenylethyleneglycol sulfate) had changed profiles in both plasma and urine but their variable importance in projection scores were opposites. These patterns must be taken into account in development of diagnostic tools.

Of the annotated metabolites, changes in the levels of 8 plasma metabolites and 23 urinary metabolites associated with diabetes in both WT and meprin βKO mice, indicating that they are independent of meprin expression or deficiency. Many of these metabolites were reported to associate with diabetic kidney disease or other diabetes complications in previous studies. These include increased plasma and urinary cortisol levels [62], increased urinary betaine excretion [66], decreased urinary galactonic acid levels [43], and increased urinary hippuric acid levels [67]. Furthermore, changes in the levels of polyphenol metabolites were observed in both WT and meprin β null mice, indicating the involvement of gut microbiota in the progression of diabetic complications [68]. Certain changes in the intestinal microbiota have been associated with early kidney disease [69]. There were similar levels of decrease in urinary excretion of pantothenic acid in both WT and meprin βKO mice.

## Conclusions

While the targets impacted by meprin metalloproteases in DN are not fully understood, previous in vitro studies have shown that proteolytic processing by meprins could impact the metabolite milieu of the kidneys. The current metabolomics data lead us to conclude that meprin β contributes to the progression of diabetic kidney injury through mediating several pathways, reflective of the diversity of the known kidney meprin substrates. This could explain why meprins are harmful in ischemia/reperfusion-induced acute kidney injury, yet some meprin isoforms are protective in DN. Additional studies, including genomics and proteomics, are desired to better understand the role that meprins plays in the pathophysiology of diabetic kidney injury.

## Additional files

Additional file 1: **Table S1.** Differentiating metabolites in plasma at 4 weeks post-STZ for analysis in the positive mode. **Table S2.** Differentiating metabolites in plasma at 4 weeks post-STZ in the negative mode **Table S3.** Differentiating metabolites in plasma at 8 weeks post-STZ in the positive mode **Table S4.** Differentiating metabolites in plasma at 8 weeks post-STZ in the negative mode **Table S5.** Differentiating metabolites in urine 4 week in positive mode **Table S6.** Differentiating metabolites in urine at 4 weeks post-STZ in the negative mode **Table S7.** Differentiating metabolites in urine at 8 weeks post-STZ for analysis in the positive mode **Table S8.** Differentiating metabolites in urine at 8 week post-STZ for analysis in negative mode. (DOCX 113 kb)

## Abbreviations

Ac-SDKP: N-acetyl-seryl-aspartyl-lysyl-proline
BBM: Brush-border membrane
CKD: Chronic kidney disease
DN: Diabetic nephropathy
ECM: Extracellular matrix
ELISA: Enzyme-linked immunosorbent assay
EQ: Equilibration
ESRD: End-stage renal disease
FC: Fold-change
HILIC: Hydrophilic interaction liquid chromatography
IL: Interleukin
KIM-1: Kidney injury molecule 1
MCP-1: Monocyte chemoattractant protein-1
NaC: Sodium citrate
NGAL: Neutrophil gelatinase-associated lipocalin
OD: Optic density
OS-9: Osteosarcoma amplified − 9
PCA: Principle components analysis
PKA: Protein kinase A
PKC: Protein kinase C
QC: Quality control
SNP: Single nucleotide polymorphisms
STZ: Streptozotocin
T2D: Type 2 diabetes
TCA: Taurocholic acid
UACR: Urinary albumin to creatinine ratio
UPLC-QTOF MS: Ultra performance liquid chromatography-quadrupole time-of flight mass spectrometry
VIP: Variable importance in projection
WT: Wild-type
βKO: Meprin beta knockout
