# Supplementary material for: Meprin β metalloproteases associated with differential metabolite profiles in the plasma and urine of mice with type 1 diabetes and diabetic nephropathy
Source: BMC Nephrol. 2019 Apr 25;20:141. doi: 10.1186/s12882-019-1313-2 (PMC6485094; doi:10.1186/s12882-019-1313-2)
Supplement: Supplementary file 1 — Table S1. Differentiating metabolites in plasma at 4 weeks post-STZ for analysis in the positive mode. Table S2. Differentiating metabolites in plasma at 4 weeks post-STZ in the negative mode Table S3. Differentiating metabolites in plasma at 8 weeks post-STZ in the positive mode Table S4. Differentiating metabolites in plasma at 8 weeks post-STZ in the negative mode Table S5. Differentiating metabolites in urine 4 week in positive mode Table S6. Differentiating metabolites in urine at 4 weeks post-STZ in the negative mode Table S7. Differentiating metabolites in urine at 8 weeks post-STZ for analysis in the positive mode Table S8. Differentiating metabolites in urine at 8 week post-STZ for analysis in negative mode. (DOCX 113 kb) [file 12882_2019_1313_MOESM1_ESM.docx]

**Table S1 Differentiating metabolites in plasma at 4 weeks post-STZ for analysis in the positive mode.**

*Exact Wilcoxon Rank Sum Test; **A positive fold change indicates median of STZ > median of NaC; ***Wilcoxon Rank Sum Test

|  | **Retention time (min)** | **Mass** | **Annotated Metabolite Name** | **Formula** | **WT** | | | **Mep βKO** | | |
| --- | --- | --- | --- | --- | --- | --- | --- | --- | --- | --- |
|  |  |  |  |  | **VIP** | **p-value*** | **FC**** | **VIP** | **p-value***** | **FC**** |
| **Meprin βKO Only** | 5.20 | 260.1849 m/z | Hexanoylcarnitine | C13H25NO4 |  |  |  | 0.5 | 0.042 | -2.2 |
|  | 2.01 | 475.3008 n | Netilmicin | C21H41N5O7 |  |  |  | 2.8 | 0.084 | 1.4 |
|  | 3.10 | 303.1310 m/z | Tyrosyl-Valine | C14H20N2O4 |  |  |  | 0.3 | 0.016 | 2 |
|  | 8.87 | 145.0502 m/z | D-1,5-Anhydrofructose | C6H10O5 |  |  |  | 0.8 | 0.01 | -3.1 |
|  |  |  | 118 Unknowns |  |  |  |  |  |  |  |
| **WT Only** | 6.68 | 515.2939 n | Taurocholic acid | C26H45NO7S | 0.7 | 0.012 | 1.6 |  |  |  |
|  | 1.04 | 243.1023 m/z | 7-Methoxy-5-prenyloxycoumarin | C15H16O4 | 0.6 | 0.042 | 2 |  |  |  |
|  |  |  | 47 Unknowns |  |  |  |  |  |  |  |
| **Both WT and Meprin βKO** | 9.72 | 162.1138 m/z | L-Carnitine | C7H15NO3 | 0.9 | 0.019 | -3.3 | 1.2 | 0.01 | -3.2 |
|  | 5.48 | 495.3330 n | LysoPC(16:0) | C24H50NO7P | 6.9 | 0.797 | -1.4 | 6.5 | 0.659 | -1.2 |
|  | 5.52 | 494.3226 m/z | LysoPC(16:1(9Z)) | C24H48NO7P | 5.1 | 0.002 | -4 | 4.8 | 0.026 | -2.3 |
|  | 7.14 | 418.1268 n | Equol 7-O-glucuronide | C21H22O9 | 0.2 | 0.029 | 2.1 | 0.5 | 0.026 | 1.7 |
|  |  |  | 52 Unknowns |  |  |  |  |  |  |  |

**Table S2 Differentiating metabolites in plasma at 4 weeks post-STZ in the negative mode**

*Exact Wilcoxon Rank Sum Test; **A positive fold change indicates median of STZ > median of NaC; ***Wilcoxon Rank Sum Test

|  | **Retention time (min)** | **Mass** | **Annotated Metabolite Name** | **Formula** | **WT** | | | **Mep βKO** | | |
| --- | --- | --- | --- | --- | --- | --- | --- | --- | --- | --- |
|  |  |  |  |  | **VIP** | **p-value*** | **FC**** | **VIP** | **p-value***** | **FC**** |
| **Meprin βKO Only** | 3.27 | 203.1152 n | L-Acetylcarnitine | C9H17NO4 |  |  |  | 1 | 0.021 | 3.1 |
|  | 1.85 | 245.0126 m/z | 3-Methoxy-4-hydroxyphenylethyleneglycol sulfate | C9H12O7S |  |  |  | 1.7 | 0.047 | 3.3 |
|  | 2.99 | 206.0818 m/z | Phenylpropionylglycine | C11H13NO3 |  |  |  | 1.1 | 0.013 | 1.3 |
|  | 2.54 | 172.0976 m/z | N-Acetylleucine | C8H15NO3 |  |  |  | 1 | 0.021 | 2 |
|  | 1.10 | 269.0450 m/z | Porric acid B | C15H12O6 |  |  |  | 0.5 | 0.021 | 3.7 |
|  |  |  | 71 Unknowns |  |  |  |  |  |  |  |
| **WT Only** | 1.71 | 263.0221 m/z | 3-Methoxy-4-hydroxyphenylethyleneglycol sulfate | C9H12O7S | 0.7 | 0.003 | 6.6 |  |  |  |
|  | 3.33 | 192.0662 m/z | 3-Methoxytyrosine | C10H13NO4 | 0.5 | 0.018 | -3.8 |  |  |  |
|  | 1.38 | 350.2084 n | 20-oxo-leukotriene B4 | C20H30O5 | 0.8 | 0.018 | 1.8 |  |  |  |
|  | 2.58 | 186.1128 m/z | N-Heptanoylglycine | C9H17NO3 | 0.6 | 0.005 | -5.2 |  |  |  |
|  | 1.12 | 349.0016 m/z | Apigenin 7-sulfate | C15H10O8S | 0.3 | 0.048 | 1.6 |  |  |  |
|  |  |  | 102 Unknowns |  |  |  |  |  |  |  |
| **Both WT and Meprin βKO** | 2.76 | 361.2011 m/z | Cortisol | C21H30O5 | 1.7 | 0.003 | 23.2 | 0.9 | 0.013 | 2.4 |
|  | 3.66 | 178.0510 m/z | Hippuric acid | C9H9NO3 | 0.9 | 0.048 | 2.4 | 1.6 | 0.013 | 2.7 |
|  | 2.89 | 172.0976 m/z | Isovalerylalanine | C8H15NO3 | 0.5 | 0.005 | -2.3 | 0.8 | 0.013 | -2.2 |
|  | 1.91 | 249.0066 m/z | 3,4-Dihydroxyphenylglycol O-sulfate | C8H10O7S | 0.5 | 0.005 | 2.4 | 0.4 | 0.036 | 1.2 |
|  | 1.60 | 477.2613 m/z | APGPR Enterostatin | C21H36N8O6 | 1.1 | 0.005 | -3 | 1.5 | 0.013 | -4 |
|  | 2.31 | 239.9968 m/z | Indole-3-carboxilic acid-O-sulphate | C9H7NO5S | 0.7 | 0.005 | 4.7 | 0.2 | 0.013 | 10.3 |
|  | 7.12 | 417.1184 m/z | Fenoprofen glucuronide | C21H22O9 | 0.7 | 0.005 | 3.4 | 1.4 | 0.013 | 3.9 |
|  |  |  | 108 Unknowns |  |  |  |  |  |  |  |

**Table S3. Differentiating metabolites in plasma at 8 weeks post-STZ in the positive mode**

*Exact Wilcoxon Rank Sum Test; **A positive fold change indicates median of STZ > median of NaC; ***Wilcoxon Rank Sum Test

|  | **Retention time (min)** | **Mass** | **Annotated Metabolite Name** | **Formula** | **WT** | | | **Mep βKO** | | |
| --- | --- | --- | --- | --- | --- | --- | --- | --- | --- | --- |
|  |  |  |  |  | **VIP** | **p-value*** | **FC**** | **VIP** | **p-value***** | **FC**** |
| **Meprin βKO Only** |  |  | 69 Unknowns |  |  |  |  |  |  |  |
| **WT Only** | 6.68 | 515.2939 n | Taurocholic acid | C26H45NO7S | 3.2 | 0.019 | 2.5 |  |  |  |
|  | 7.49 | 243.0864 n | Cytidine | C9H13N3O5 | 0.2 | 0.045 | 1.9 |  |  |  |
|  | 6.68 | 152.0581 m/z | Guanine | C5H5N5O | 0.3 | 0.019 | 2.7 |  |  |  |
|  | 6.19 | 376.1386 n | Riboflavin | C17H20N4O6 | 0.4 | 0.019 | 2.5 |  |  |  |
|  | 2.26 | 153.0434 n | 3-Aminosalicylic acid | C7H7NO3 | 0.9 | 0.045 | 3.6 |  |  |  |
|  | 5.52 | 494.3226 m/z | LysoPC(16:1(9Z)) | C24H48NO7P | 3 | 0.019 | -3.6 |  |  |  |
|  | 3.79 | 394.2934 m/z | 9'-Carboxy-gamma-chromanol | C23H36O4 | 0.5 | 0.019 | 2.5 |  |  |  |
|  | 1.04 | 243.1023 m/z | 7-Methoxy-5-prenyloxycoumarin | C15H16O4 | 0.4 | 0.019 | 2 |  |  |  |
|  | 6.81 | 140.0713 m/z | 5-Acetyl-2,4-dimethyloxazole | C7H9NO2 | 0.4 | 0.003 | 3.1 |  |  |  |
|  |  |  | 236 Unknowns |  |  |  |  |  |  |  |
| **Both WT and Meprin βKO** | 5.48 | 495.3330 n | LysoPC(16:0) | C24H50NO7P | 5.2 | 0.943 | 1 | 5.2 | 0.849 | 1.3 |
|  | 2.01 | 475.3008 n | Netilmicin | C21H41N5O7 | 2.7 | 0.127 | 3.9 | 3 | 0.161 | 1.5 |
|  | 3.10 | 303.1310 m/z | Tyrosyl-Valine | C14H20N2O4 | 0.3 | 0.019 | 3.4 | 0.3 | 0.033 | 1.9 |
|  | 2.58 | 740.4641 m/z | Ophiopogonin C' | C39H62O12 | 3.4 | 0.127 | 2.8 | 3.9 | 0.161 | 1.3 |
|  | 7.14 | 418.1268 n | Equol 7-O-glucuronide | C21H22O9 | 0.3 | 0.002 | 4.5 | 0.4 | 0.02 | 3.2 |
|  |  |  | 85 Unknowns |  |  |  |  |  |  |  |

**Table S4. Differentiating metabolites in plasma at 8 weeks post-STZ in the negative mode**

*Exact Wilcoxon Rank Sum Test; **A positive fold change indicates median of STZ > median of NaC; ***Wilcoxon Rank Sum Test

|  | **Retention time (min)** | **Mass (m/z)** | **Annotated Metabolite Name** | **Formula** | **WT** | | | **Mep βKO** | | |
| --- | --- | --- | --- | --- | --- | --- | --- | --- | --- | --- |
|  |  |  |  |  | **VIP** | **p-value*** | **FC**** | **VIP** | **p-value***** | **FC**** |
| **Meprin βKO Only** | 2.89 | 172.0976 | Isovalerylalanine | C8H15NO3 |  |  |  | 0.7 | 0.016 | -2.5 |
|  |  |  | 77 Unknowns |  |  |  |  |  |  |  |
| **WT Only** | 1.71 | 263.0221 | 3-Methoxy-4-hydroxyphenylethyleneglycol sulfate | C9H12O7S | 0.5 | 0.01 | 9.1 |  |  |  |
|  | 1.18 | 212.0032 | Indoxyl sulfate | C8H7NO4S | 5.3 | 0.003 | 2.1 |  |  |  |
|  | 2.99 | 206.0818 | Phenylpropionylglycine | C11H13NO3 | 0.7 | 0.01 | 1.4 |  |  |  |
|  | 1.20 | 132.0448 | 2-Phenylglycine | C8H9NO2 | 0.3 | 0.003 | 2.1 |  |  |  |
|  | 2.54 | 172.0976 | N-Acetylleucine | C8H15NO3 | 1 | 0.005 | 2.2 |  |  |  |
|  | 2.58 | 186.1128 | N-Heptanoylglycine | C9H17NO3 | 0.2 | 0.03 | -2.1 |  |  |  |
|  | 1.12 | 349.0016 | Apigenin 7-sulfate | C15H10O8S | 0.4 | 0.018 | 1.9 |  |  |  |
|  |  |  | 122 Unknowns |  |  |  |  |  |  |  |
| **Both WT and Meprin βKO** | 2.76 | 361.2011 | Cortisol | C21H30O5 | 0.9 | 0.003 | 4.2 | 0.8 | 0.026 | 4.4 |
|  | 1.85 | 245.0126 | 3-Methoxy-4-hydroxyphenylethyleneglycol sulfate | C9H12O7S | 0.7 | 0.03 | 3.2 | 0.9 | 0.042 | 2.7 |
|  | 3.66 | 178.0510 | Hippuric acid | C9H9NO3 | 1.2 | 0.003 | 3.2 | 1.2 | 0.012 | 2.5 |
|  | 1.91 | 249.0066 | 3,4-Dihydroxyphenylglycol O-sulfate | C8H10O7S | 0.4 | 0.005 | 2.2 | 0.5 | 0.016 | 1.8 |
|  | 1.60 | 477.2613 | APGPR Enterostatin | C21H36N8O6 | 0.9 | 0.03 | -1.7 | 1.3 | 0.042 | -1.7 |
|  | 2.31 | 239.9968 | Indole-3-carboxilic acid-O-sulphate | C9H7NO5S | 0.5 | 0.003 | 6.2 | 0.1 | 0.01 | 935.1 |
|  | 7.12 | 17.1184 | Fenoprofen glucuronide | C21H22O9 | 0.7 | 0.003 | 3.6 | 0.8 | 0.01 | 3.1 |
|  |  |  | 113 Unknowns |  |  |  |  |  |  |  |

**Table S5. Differentiating metabolites in urine 4 week in positive mode**

*Exact Wilcoxon Rank Sum Test; **A positive fold change indicates median of STZ > median of NaC; ***p-value was not calculated due to small sample size

|  | **Retention time (min)** | **Mass** | **Annotated Metabolite Name** | **Formula** | **WT** | | | **Mep βKO** | | |
| --- | --- | --- | --- | --- | --- | --- | --- | --- | --- | --- |
|  |  |  |  |  | **VIP** | **p-value*** | **FC**** | **VIP** | **p-value***** | **FC**** |
| **Meprin βKO Only** | 6.19 | 376.1386 n | Riboflavin | C17H20N4O6 |  |  |  | 2.5 |  | -1.3 |
|  |  |  | 7 Unknowns |  |  |  |  |  |  |  |
| **WT Only** | 3.18 | 166.0506 m/z | 4-Pyridoxic acid | C8H9NO4 | 0.4 | 0.034 | 1.4 |  |  |  |
|  | 6.68 | 515.2939 n | Taurocholic acid | C26H45NO7S | 0.5 | 0.003 | 4 |  |  |  |
|  | 9.72 | 162.1138 m/z | L-Carnitine | C7H15NO3 | 3.7 | 0.106 | -2.1 |  |  |  |
|  | 6.68 | 152.0581 m/z | Guanine | C5H5N5O | 0.4 | 0.011 | 1.4 |  |  |  |
|  | 1.75 | 301.1268 m/z | Guanosine | C10H13N5O5 | 1.3 | 0.003 | 1.6 |  |  |  |
|  | 9.79 | 97.0768 m/z | 3-Amino-2-piperidone | C5H10N2O | 0.6 | 0.003 | -1.6 |  |  |  |
|  | 1.56 | 162.0555 m/z | Hippuric acid | C9H9NO3 | 1.4 | 0.034 | -2 |  |  |  |
|  | 6.33 | 189.0433 n | Kynurenic acid | C10H7NO3 | 1.7 | 0.003 | -1.5 |  |  |  |
|  | 1.36 | 91.0548 m/z | p-Cresol | C7H8O | 0.6 | 0.003 | 1.8 |  |  |  |
|  | 1.20 | 105.0340 m/z | Benzoic acid | C7H6O2 | 0.2 | 0.011 | 1.7 |  |  |  |
|  | 9.79 | 229.0890 n | Ergothioneine | C9H15N3O2S | 0.3 | 0.003 | 2.1 |  |  |  |
|  | 8.89 | 282.1203 m/z | 1-Methyladenosine | C11H15N5O4 | 2.1 | 0.604 | -1.2 |  |  |  |
|  | 2.09 | 153.0667 m/z | N1-Methyl-4-pyridone-3-carboxamide | C7H8N2O2 | 5.1 | 0.003 | 1.6 |  |  |  |
|  | 5.52 | 494.3226 m/z | LysoPC(16:1) | C24H48NO7P | 0.5 | 0.003 | 2.9 |  |  |  |
|  | 3.79 | 394.2934 m/z | 9'-Carboxy-gamma-chromanol | C23H36O4 | 0.2 | 0.011 | 1.7 |  |  |  |
|  | 9.47 | 248.1495 m/z | Hydroxybutyrylcarnitine | C11H21NO5 | 1 | 0.003 | -6.2 |  |  |  |
|  | 10.29 | 337.0803 n | 3-Indole carboxylic acid glucuronide | C15H15NO8 | 2.4 | 0.034 | -1.5 |  |  |  |
|  | 3.81 | 251.1028 m/z | Tyrosyl-Serine | C12H16N2O5 | 0.5 | 0.034 | 1.3 |  |  |  |
|  | 3.10 | 303.1310 m/z | Tyrosyl-Valine | C14H20N2O4 | 1.8 | 0.003 | 3.3 |  |  |  |
|  | 8.70 | 253.1168 m/z | Valyl-Hydroxyproline | C10H18N2O4 | 0.9 | 0.02 | -1.8 |  |  |  |
|  | 1.43 | 231.1710 m/z | Neoherculin | C16H25NO | 0.1 | 0.003 | 1.4 |  |  |  |
|  | 9.79 | 143.0823 m/z | 4-Acetamido-2-aminobutanoic acid | C6H12N2O3 | 1.9 | 0.003 | -1.6 |  |  |  |
|  | 10.75 | 266.1612 m/z | Prenyl glucoside | C11H20O6 | 1.2 | 0.011 | -2 |  |  |  |
|  | 2.01 | 219.1749 m/z | Isolubimin | C15H24O2 | 0.4 | 0.003 | 1.8 |  |  |  |
|  | 7.14 | 243.1024 m/z | Wyerol | C15H16O4 | 1.2 | 0.011 | 1.5 |  |  |  |
|  | 1.38 | 364.1883 n | 3b,8b-Dihydroxy-6b-angeloyloxy-7(11)-eremophilen-12,8-olide | C20H28O6 | 1.8 | 0.003 | 2.7 |  |  |  |
|  | 8.36 | 430.0906 n | Daidzein 7-O-glucuronide | C21H18O10 | 1.1 | 0.02 | 1.4 |  |  |  |
|  | 7.08 | 444.1060 n | Formononetin 7-O-glucuronide | C22H20O10 | 1.4 | 0.034 | 1.5 |  |  |  |
|  | 2.39 | 242.0119 m/z | Indole-3-carboxilic acid-O-sulphate | C9H7NO5S | 1.7 | 0.003 | 2.7 |  |  |  |
|  |  |  | 2,475 Unknowns |  |  |  |  |  |  |  |
| **Both WT and Meprin βKO** | 8.24 | 117.0798 n | Betaine | C5H11NO2 | 4.1 | 0.006 | 2 | 3.2 |  | 1.5 |
|  | 11.70 | 257.1036 n | Glycerophosphocholine | C8H20NO6P | 3.9 | 0.003 | -6.9 | 2.6 |  | -3 |
|  | 10.00 | 141.0667 m/z | Nicotinic acid | C6H5NO2 | 3.3 | 0.003 | -5.9 | 3.8 |  | -3.1 |
|  | 2.26 | 153.0434 n | 3-Aminosalicylic acid | C7H7NO3 | 6.1 | 0.003 | 1.7 | 5.6 |  | 1.2 |
|  | 12.54 | 203.1510 m/z | Symmetric dimethylarginine | C8H18N4O2 | 3.5 | 0.003 | -2.6 | 4.3 |  | -2.3 |
|  | 10.77 | 217.1304 m/z | N-a-Acetyl-L-arginine | C8H16N4O3 | 2.9 | 0.003 | -3.3 | 2.4 |  | -1.6 |
|  | 1.04 | 243.1023 m/z | 7-Methoxy-5-prenyloxycoumarin | C15H16O4 | 2.6 | 0.003 | 2.5 | 2.3 |  | 1.6 |
|  | 1.36 | 279.1573 m/z | Lotaustralin | C11H19NO6 | 2.5 | 0.003 | 1.8 | 2.4 |  | 1.6 |
|  | 6.81 | 140.0713 m/z | 5-Acetyl-2,4-dimethyloxazole | C7H9NO2 | 2.3 | 0.003 | 1.7 | 2.6 |  | 1.7 |
|  | 7.14 | 418.1268 n | Equol 7-O-glucuronide | C21H22O9 | 3.2 | 0.003 | 1.6 | 4.1 |  | 1.5 |
|  | 9.70 | 302.0645 n | Pyrogallol-2-O-glucuronide | C12H14O9 | 1.7 | 0.003 | -4.8 | 2.2 |  | -2.6 |
|  | 10.83 | 350.1022 m/z | S-(Formylmethyl)glutathione | C12H19N3O7S | 1.4 | 0.003 | -31 | 2.7 |  | -5.6 |
|  |  |  | 96 Unknowns |  |  |  |  |  |  |  |

**Table S6. Differentiating metabolites in urine at 4 weeks post-STZ in the negative mode**

*Exact Wilcoxon Rank Sum Test; **A positive fold change indicates median of STZ > median of NaC; ***p-value was not calculated due to small sample size

|  | **Retention time (min)** | **Mass** | **Annotated Metabolite Name** | **Formula** | **WT** | | | **Mep βKO** | | |
| --- | --- | --- | --- | --- | --- | --- | --- | --- | --- | --- |
|  |  |  |  |  | **VIP** | **p-value*** | **FC**** | **VIP** | **p-value***** | **FC**** |
| **Meprin βKO Only** | 1.85 | 245.0126 m/z | 3-Methoxy-4-hydroxyphenylethyleneglycol sulfate | C9H12O7S |  |  |  | 14.2 |  | 1.3 |
|  | 1.85 | 165.0550 m/z | 4-Methoxyphenylacetic acid | C9H10O3 |  |  |  | 2.3 |  | 1.4 |
|  |  |  | 14 Unknowns |  |  |  |  |  |  |  |
| **WT Only** | 2.76 | 361.2011 m/z | Cortisol | C21H30O5 | 10.5 | 0.003 | 54.9 |  |  |  |
|  | 12.84 | 193.0343 m/z | D-Glucuronic acid | C6H10O7 | 0.5 | 0.034 | -1.4 |  |  |  |
|  | 5.24 | 157.0362 m/z | Allantoin | C4H6N4O3 | 1.5 | 0.003 | 1.3 |  |  |  |
|  | 7.61 | 113.0240 m/z | Monoethyl malonic acid | C5H8O4 | 0.3 | 0.006 | 1.4 |  |  |  |
|  | 3.51 | 204.0663 m/z | Indolelactic acid | C11H11NO3 | 5.9 | 0.011 | -1.7 |  |  |  |
|  | 1.18 | 212.0032 m/z | Indoxyl sulfate | C8H7NO4S | 8.5 | 0.825 | 1.4 |  |  |  |
|  | 1.02 | 174.0555 m/z | Phenylacetylglycine | C10H11NO3 | 0.4 | 0.003 | -3.4 |  |  |  |
|  | 2.99 | 206.0818 m/z | Phenylpropionylglycine | C11H13NO3 | 4.8 | 0.011 | -2.4 |  |  |  |
|  | 5.72 | 206.0457 m/z | 4-(2-Aminophenyl)-2,4-dioxobutanoic acid | C10H9NO4 | 0.9 | 0.006 | -1.6 |  |  |  |
|  | 3.33 | 192.0662 m/z | 3-Methoxytyrosine | C10H13NO4 | 2.8 | 0.148 | -1.7 |  |  |  |
|  | 1.91 | 249.0066 m/z | 3,4-Dihydroxyphenylglycol O-sulfate | C8H10O7S | 2.3 | 0.199 | 1.4 |  |  |  |
|  | 2.82 | 167.0346 m/z | 5-Methoxysalicylic acid | C8H8O4 | 2 | 0.94 | -1 |  |  |  |
|  | 3.64 | 187.0068 m/z | p-Cresol sulfate | C7H8O4S | 2.3 | 0.825 | 1.2 |  |  |  |
|  | 1.51 | 261.0065 m/z | Homovanillic acid sulfate | C9H10O7S | 3.5 | 0.148 | -1.3 |  |  |  |
|  | 2.14 | 242.9963 m/z | Homovanillic acid sulfate | C9H10O7S | 2.1 | 0.414 | -1.9 |  |  |  |
|  | 3.01 | 261.0073 m/z | Homovanillic acid sulfate | C9H10O7S | 8.3 | 0.02 | 4.1 |  |  |  |
|  | 3.87 | 261.0072 m/z | Homovanillic acid sulfate | C9H10O7S | 5.8 | 0.003 | -1.3 |  |  |  |
|  | 6.53 | 186.0767 m/z | 2-Keto-6-acetamidocaproate | C8H13NO4 | 1 | 0.003 | -1.9 |  |  |  |
|  | 1.38 | 350.2084 n | 20-oxo-leukotriene B4 | C20H30O5 | 2.1 | 0.003 | 3.3 |  |  |  |
|  | 2.58 | 186.1128 m/z | N-Heptanoylglycine | C9H17NO3 | 4.7 | 0.011 | -3.9 |  |  |  |
|  | 1.77 | 319.1554 m/z | Omega-Carboxy-trinor-leukotriene B4 | C18H26O6 | 0.2 | 0.034 | 1.5 |  |  |  |
|  | 4.39 | 243.0616 m/z | Ng-L-Glutamyl-L-aspartic acid | C9H14N2O7 | 1.6 | 0.011 | -1.5 |  |  |  |
|  | 1.02 | 285.0744 m/z | Heliannone C | C16H14O5 | 0.5 | 0.011 | 1.8 |  |  |  |
|  | 1.34 | 227.1279 m/z | Dibutyl malate | C12H22O5 | 1.2 | 0.006 | 1.7 |  |  |  |
|  | 1.10 | 269.0450 m/z | Porric acid B | C15H12O6 | 3.3 | 0.003 | -3.7 |  |  |  |
|  | 2.95 | 123.0445 m/z | 2-Acetyl-3-methylfuran | C7H8O2 | 2 | 0.011 | 1.4 |  |  |  |
|  | 5.85 | 387.2007 m/z | 9,13-Dihydroxy-4-megastigmen-3-one 9-glucoside | C19H32O8 | 1.6 | 0.003 | -22.9 |  |  |  |
|  | 3.62 | 515.0857 m/z | 3,5-Dihydroxy-3',4'-dimethoxy-6,7-methylenedioxyflavone 3-glucuronide | C24H22O14 | 3.7 | 0.33 | 1.2 |  |  |  |
|  | 6.23 | 387.1653 m/z | beta-D-Glucopyranosyl-11-hydroxyjasmonic acid | C18H28O9 | 2.2 | 0.71 | -1.5 |  |  |  |
|  | 8.29 | 611.1628 m/z | Aromadendrin 3,7-diglucoside | C27H32O16 | 1.3 | 0.02 | 6.9 |  |  |  |
|  | 6.96 | 559.1129 m/z | Cicerin 7-(6-malonylglucoside) | C26H26O15 | 3.5 | 0.003 | 3.7 |  |  |  |
|  | 3.45 | 211.0616 m/z | 3-Hydroxy-4-methoxyphenyllactic acid | C10H12O5 | 0.5 | 0.006 | -1.5 |  |  |  |
|  | 1.62 | 258.9915 m/z | Caffeic acid 4-sulfate | C9H8O7S | 3.8 | 0.011 | -1.4 |  |  |  |
|  | 4.51 | 258.9912 m/z | Caffeic acid 4-sulfate | C9H8O7S | 2.5 | 0.003 | -2.7 |  |  |  |
|  | 1.87 | 273.0068 m/z | Isoferulic acid 3-sulfate | C10H10O7S | 4.9 | 0.003 | -1.6 |  |  |  |
|  | 7.37 | 449.1086 m/z | Phloretin 2'-O-glucuronide | C21H22O11 | 1.2 | 0.003 | 3.6 |  |  |  |
|  | 1.14 | 167.0706 m/z | 1,3,5-Trimethoxybenzene | C9H12O3 | 1.5 | 0.003 | -1.9 |  |  |  |
|  | 2.39 | 216.9806 m/z | 4-hydroxybenzoic acid-4-O-sulphate | C7H6O6S | 2.5 | 0.003 | -1.3 |  |  |  |
|  | 3.27 | 230.9974 m/z | Hydroxymethoxyphenylcarboxylic acid-O-sulphate | C8H10O7S | 4.6 | 0.33 | 1.1 |  |  |  |
|  |  |  | 2330 Unknowns |  |  |  |  |  |  |  |
| **Both WT and Meprin βKO** | 1.26 | 175.0243 m/z | D-Glucuronic acid | C6H10O7 | 3.5 | 0.006 | 1.5 | 4.4 |  | 1.2 |
|  | 3.92 | 218.1028 m/z | Pantothenic acid | C9H17NO5 | 2.8 | 0.003 | -1.4 | 4.7 |  | -1.5 |
|  | 1.71 | 263.0221 m/z | 3-Methoxy-4-hydroxyphenylethyleneglycol sulfate | C9H12O7S | 3.6 | 0.003 | 1.6 | 3.3 |  | 1.2 |
|  | 12.29 | 195.0509 m/z | Galactonic acid | C6H12O7 | 4.3 | 0.003 | -3.1 | 5.2 |  | -2.5 |
|  | 2.76 | 179.0345 m/z | 4-Hydroxyphenylpyruvic acid | C9H8O4 | 2.5 | 0.003 | 2.6 | 2.2 |  | 4.8 |
|  | 3.66 | 178.0510 m/z | Hippuric acid | C9H9NO3 | 9.4 | 0.003 | 1.6 | 10.3 |  | 1.2 |
|  | 5.42 | 174.0764 m/z | N-Carboxyethyl-g-aminobutyric acid | C7H13NO4 | 1.5 | 0.003 | -3.3 | 2.2 |  | -2.8 |
|  | 4.65 | 128.0346 m/z | 1-Pyrroline-4-hydroxy-2-carboxylate | C5H7NO3 | 2.5 | 0.003 | -13.2 | 2.6 |  | -3.6 |
|  | 7.26 | 160.0399 m/z | Indole-3-carboxylic acid | C9H7NO2 | 2.4 | 0.003 | -4.8 | 2.3 |  | -2.2 |
|  | 2.78 | 194.0456 m/z | 3-Hydroxyhippuric acid | C9H9NO4 | 3.9 | 0.106 | 1.4 | 6.3 |  | 1.1 |
|  | 2.54 | 172.0976 m/z | N-Acetylleucine | C8H15NO3 | 7.5 | 0.02 | 2.2 | 7 |  | 2 |
|  | 3.53 | 415.1963 m/z | Ethyl 7-epi-12-hydroxyjasmonate glucoside | C20H32O9 | 2 | 0.604 | -1.2 | 4.4 |  | 1.3 |
|  | 8.83 | 202.0716 m/z | Avenic acid B | C8H15NO6 | 1.4 | 0.003 | -7.3 | 2.5 |  | -4.8 |
|  | 14.16 | 351.0566 m/z | a-L-threo-4-Hex-4-enopyranuronosyl-D-galacturonic acid | C12H16O12 | 2.8 | 0.003 | -172.7 | 3.9 |  | -20.8 |
|  | 2.76 | 258.9916 m/z | Caffeic acid 3-sulfate | C9H8O7S | 6.6 | 0.003 | 2.4 | 5.8 |  | 4.2 |
|  | 7.41 | 445.0772 m/z | Genistein 5-O-glucuronide | C21H18O11 | 3.2 | 0.003 | 2.3 | 2.9 |  | 1.2 |
|  | 8.44 | 308.0773 m/z | Indoxylglucuronide | C14H15NO7 | 1.8 | 0.034 | -2.5 | 3.5 |  | -2 |
|  | 2.31 | 239.9968 m/z | Indole-3-carboxilic acid-O-sulphate | C9H7NO5S | 3.9 | 0.02 | 1.6 | 2.4 |  | 1.8 |
|  | 1.26 | 254.9813 m/z | Ascorbic acid-2-sulfate | C6H8O9S | 8.8 | 0.006 | 1.6 | 11.1 |  | 1.2 |
|  | 7.12 | 417.1184 m/z | Fenoprofen glucuronide | C21H22O9 | 6.7 | 0.003 | 1.6 | 10 |  | 1.5 |
|  |  |  | 98 Unknowns |  |  |  |  |  |  |  |

**Table S7. Differentiating metabolites in urine at 8 weeks post-STZ for analysis in the positive mode**

*Exact Wilcoxon Rank Sum Test; **A positive fold change indicates median of STZ > median of NaC

|  | **Retention Time (min)** | **Mass** | **Annotated Metabolite Name** | **Formula** | **WT** | | | ***Mep1b*^-/-^** | | |
| --- | --- | --- | --- | --- | --- | --- | --- | --- | --- | --- |
|  |  |  |  |  | **VIP** | **p-value*** | **FC**** | **VIP** | **p-value*** | **FC**** |
| **Meprin βKO Only** | 3.18 | 166.0506 m/z | 4-Pyridoxic acid | C8H9NO4 |  |  |  | 0.5 | 0.03 | 1.7 |
|  | 6.19 | 376.1386 n | Riboflavin | C17H20N4O6 |  |  |  | 2.7 | 0.126 | -1.5 |
|  | 8.89 | 282.1203 m/z | 1-Methyladenosine | C11H15N5O4 |  |  |  | 2.6 | 0.792 | -1.1 |
|  | 3.16 | 215.0860 m/z | Methyl bisnorbiotinyl ketone | C9H14N2O2S |  |  |  | 1.6 | 0.017 | 1.4 |
|  | 10.29 | 161.0483 n | 3-Formyl-6-hydroxyindole | C9H7NO2 |  |  |  | 1.2 | 0.017 | 3.6 |
|  | 3.55 | 135.0808 m/z | 2-Phenylpropanal | C9H10O |  |  |  | 1.1 | 0.03 | 1.7 |
|  | 2.01 | 219.1749 m/z | Isolubimin | C15H24O2 |  |  |  | 0.5 | 0.03 | 1.5 |
|  | 7.08 | 444.1060 n | Formononetin 7-O-glucuronide | C22H20O10 |  |  |  | 2.3 | 0.004 | 1.7 |
|  | 6.31 | 461.1081 m/z | Glycitein 7-O-glucuronide | C22H20O11 |  |  |  | 1.5 | 0.004 | 1.6 |
|  |  |  | 120 Unknowns |  |  |  |  |  |  |  |
| **WT Only** | 6.68 | 515.2939 n | Taurocholic acid | C26H45NO7S | 0.3 | 0.002 | 2 |  |  |  |
|  | 1.75 | 301.1268 m/z | Guanosine | C10H13N5O5 | 1.1 | 0.006 | 1.4 |  |  |  |
|  | 9.79 | 97.0768 m/z | 3-Amino-2-piperidone | C5H10N2O | 0.6 | 0.002 | -1.6 |  |  |  |
|  | 6.33 | 189.0433 n | Kynurenic acid | C10H7NO3 | 1.9 | 0.003 | -1.5 |  |  |  |
|  | 1.36 | 91.0548 m/z | p-Cresol | C7H8O | 0.5 | 0.002 | 1.5 |  |  |  |
|  | 1.20 | 105.0340 m/z | Benzoic acid | C7H6O2 | 0.3 | 0.002 | 1.7 |  |  |  |
|  | 9.79 | 229.0890 n | Ergothioneine | C9H15N3O2S | 0.3 | 0.002 | 2.1 |  |  |  |
|  | 5.48 | 495.3330 n | LysoPC(16:0) | C24H50NO7P | 1 | 0.03 | -1.5 |  |  |  |
|  | 5.52 | 494.3226 m/z | LysoPC(16:1) | C24H48NO7P | 0.3 | 0.03 | 1.6 |  |  |  |
|  | 8.89 | 150.0779 m/z | 1-Methyladenine | C6H7N5 | 1.6 | 0.03 | 1.2 |  |  |  |
|  | 3.79 | 394.2934 m/z | 9'-Carboxy-gamma-chromanol | C23H36O4 | 0.2 | 0.003 | 1.6 |  |  |  |
|  | 3.81 | 251.1028 m/z | Tyrosyl-Serine | C12H16N2O5 | 0.5 | 0.019 | 1.3 |  |  |  |
|  | 3.10 | 303.1310 m/z | Tyrosyl-Valine | C14H20N2O4 | 1.4 | 0.006 | 2.3 |  |  |  |
|  | 1.43 | 231.1710 m/z | Neoherculin | C16H25NO | 0.1 | 0.03 | 1.3 |  |  |  |
|  | 9.79 | 143.0823 m/z | 4-Acetamido-2-aminobutanoic acid | C6H12N2O3 | 2 | 0.002 | -1.6 |  |  |  |
|  | 10.75 | 266.1612 m/z | Prenyl glucoside | C11H20O6 | 2 | 0.002 | -3.1 |  |  |  |
|  | 1.04 | 243.1023 m/z | 7-Methoxy-5-prenyloxycoumarin | C15H16O4 | 2.4 | 0.002 | 1.9 |  |  |  |
|  | 1.20 | 594.1319 m/z | Quercetin 3-galactoside 7-rhamnoside | C27H30O16 | 0.5 | 0.002 | 1.6 |  |  |  |
|  | 1.12 | 351.0172 m/z | Apigenin 7-sulfate | C15H10O8S | 0.6 | 0.045 | -1.6 |  |  |  |
|  | 6.81 | 140.0713 m/z | 5-Acetyl-2,4-dimethyloxazole | C7H9NO2 | 2.2 | 0.002 | 1.7 |  |  |  |
|  | 9.70 | 302.0645 n | Pyrogallol-2-O-glucuronide | C12H14O9 | 1.5 | 0.006 | -3.5 |  |  |  |
|  |  |  | 1,761 Unknowns |  |  |  |  |  |  |  |
| **Both WT and Meprin βKO** | 8.24 | 117.0798 n | Betaine | C5H11NO2 | 2.2 | 0.045 | 1.3 | 3.4 | 0.537 | 1.3 |
|  | 9.72 | 162.1138 m/z | L-Carnitine | C7H15NO3 | 4.6 | 0.002 | -2.4 | 6.2 | 0.03 | -1.7 |
|  | 11.70 | 257.1036 n | Glycerophosphocholine | C8H20NO6P | 4 | 0.002 | -4.8 | 4.1 | 0.017 | -2.2 |
|  | 5.20 | 260.1849 m/z | Hexanoylcarnitine | C13H25NO4 | 0.3 | 0.019 | -1.5 | 0.5 | 0.03 | -1.2 |
|  | 6.15 | 160.1335 m/z | DL-2-Aminooctanoic acid | C8H17NO2 | 1 | 0.019 | -1.5 | 1.7 | 0.03 | 1.6 |
|  | 10.00 | 141.0667 m/z | Nicotinic acid | C6H5NO2 | 3.3 | 0.002 | -3.6 | 3.9 | 0.004 | -2.4 |
|  | 5.89 | 160.0764 m/z | 5-Hydroxytryptophol | C10H11NO2 | 0.5 | 0.006 | -1.2 | 0.4 | 0.017 | -1 |
|  | 2.26 | 153.0434 n | 3-Aminosalicylic acid | C7H7NO3 | 6.2 | 0.002 | 1.6 | 6.4 | 0.004 | 1.4 |
|  | 12.54 | 203.1510 m/z | Symmetric dimethylarginine | C8H18N4O2 | 2.8 | 0.019 | -1.5 | 3.8 | 0.329 | -1.4 |
|  | 2.09 | 153.0667 m/z | N1-Methyl-4-pyridone-3-carboxamide | C7H8N2O2 | 5.2 | 0.002 | 1.6 | 4.7 | 0.009 | 1.3 |
|  | 10.77 | 217.1304 m/z | N-a-Acetyl-L-arginine | C8H16N4O3 | 2.2 | 0.019 | -1.8 | 2 | 0.329 | -1.1 |
|  | 9.47 | 248.1495 m/z | Hydroxybutyrylcarnitine | C11H21NO5 | 1.1 | 0.002 | -6.5 | 1.7 | 0.004 | -5.4 |
|  | 1.36 | 279.1573 m/z | Lotaustralin | C11H19NO6 | 2.2 | 0.003 | 1.5 | 2.4 | 0.052 | 1.3 |
|  | 7.14 | 243.1024 m/z | Wyerol | C15H16O4 | 1.5 | 0.011 | 1.6 | 2.1 | 0.004 | 1.4 |
|  | 1.38 | 364.1883 n | 3b,8b-Dihydroxy-6b-angeloyloxy-7(11)-eremophilen-12,8-olide | C20H28O6 | 1.6 | 0.003 | 2.1 | 1.5 | 0.004 | 1.6 |
|  | 8.87 | 145.0502 m/z | D-1,5-Anhydrofructose | C6H10O5 | 0.5 | 0.003 | -2.2 | 1.1 | 0.004 | -2.3 |
|  | 8.36 | 430.0906 n | Daidzein 7-O-glucuronide | C21H18O10 | 1.4 | 0.03 | 1.5 | 2.1 | 0.004 | 1.3 |
|  | 7.14 | 418.1268 n | Equol 7-O-glucuronide | C21H22O9 | 3.5 | 0.003 | 1.7 | 4.6 | 0.004 | 1.5 |
|  | 2.39 | 242.0119 m/z | Indole-3-carboxilic acid-O-sulphate | C9H7NO5S | 1.8 | 0.002 | 2.4 | 0.6 | 0.004 | 3.3 |
|  | 10.83 | 350.1022 m/z | S-(Formylmethyl)glutathione | C12H19N3O7S | 1.2 | 0.002 | -8.4 | 2.6 | 0.126 | -2.3 |
|  |  |  | 641 Unknowns |  |  |  |  |  |  |  |

**Table S8. Differentiating metabolites in urine at 8 week post-STZ for analysis in the negative mode.**

*Exact Wilcoxon Rank Sum Test; **A positive fold change indicates median of STZ > median of NaC

|  | **Retention time (min)** | **Mass** | **Annotated Metabolite Name** | **Formula** | **WT** | | | **Mep βKO** | | |
| --- | --- | --- | --- | --- | --- | --- | --- | --- | --- | --- |
|  |  |  |  |  | **VIP** | **p-value*** | **FC**** | **VIP** | **p-value*** | **FC**** |
| **Meprin βKO Only** | 3.27 | 203.1152 n | L-Acetylcarnitine | C9H17NO4 |  |  |  | 2.5 | 0.429 | 1.2 |
|  | 3.92 | 218.1028 m/z | Pantothenic acid | C9H17NO5 |  |  |  | 4.1 | 0.017 | -1.7 |
|  | 3.98 | 207.0521 n | 4-(2-Aminophenyl)-2,4-dioxobutanoic acid | C10H9NO4 |  |  |  | 2.9 | 0.126 | -1.4 |
|  | 2.82 | 167.0346 m/z | 5-Methoxysalicylic acid | C8H8O4 |  |  |  | 3.8 | 0.082 | 1.1 |
|  | 2.78 | 194.0456 m/z | 3-Hydroxyhippuric acid | C9H9NO4 |  |  |  | 5.3 | 0.126 | 1.3 |
|  | 1.51 | 261.0065 m/z | Homovanillic acid sulfate | C9H10O7S |  |  |  | 3.9 | 0.429 | -1.2 |
|  | 1.77 | 319.1554 m/z | Omega-Carboxy-trinor-leukotriene B4 | C18H26O6 |  |  |  | 0.4 | 0.009 | 1.7 |
|  | 3.62 | 515.0857 m/z | 3,5-Dihydroxy-3',4'-dimethoxy-6,7-methylenedioxyflavone 3-glucuronide | C24H22O14 |  |  |  | 5.6 | 0.004 | -3.7 |
|  | 1.62 | 258.9915 m/z | Caffeic acid 4-sulfate | C9H8O7S |  |  |  | 3.3 | 1 | 1.1 |
|  | 7.08 | 443.0980 m/z | Formononetin 7-O-glucuronide | C22H20O10 |  |  |  | 1.3 | 0.009 | 1.7 |
|  | 1.87 | 273.0068 m/z | Isoferulic acid 3-sulfate | C10H10O7S |  |  |  | 3.1 | 0.792 | -1.2 |
|  | 8.44 | 308.0773 m/z | Indoxylglucuronide | C14H15NO7 |  |  |  | 3.2 | 0.004 | -1.7 |
|  | 2.39 | 216.9806 m/z | 4-hydroxybenzoic acid-4-O-sulphate | C7H6O6S |  |  |  | 2.2 | 0.537 | 1.1 |
|  | 1.73 | 242.9966 m/z | 3-(3,5-dihydroxyphenyl)-1-propanoic acid sulphate | C9H10O7S |  |  |  | 3.8 | 0.177 | 1.3 |
|  |  |  | 244 Unknowns |  |  |  |  |  |  |  |
| **WT Only** | 6.96 | 176.0314 n | Ascorbic acid | C6H8O6 | 1 | 0.002 | 1.5 |  |  |  |
|  | 2.76 | 361.2011 m/z | Cortisol | C21H30O5 | 8.2 | 0.002 | 21.6 |  |  |  |
|  | 5.24 | 157.0362 m/z | Allantoin | C4H6N4O3 | 1.8 | 0.002 | 1.4 |  |  |  |
|  | 7.61 | 113.0240 m/z | Monoethyl malonic acid | C5H8O4 | 0.4 | 0.011 | 1.9 |  |  |  |
|  | 2.76 | 179.0345 m/z | 4-Hydroxyphenylpyruvic acid | C9H8O4 | 1.7 | 0.003 | 2.3 |  |  |  |
|  | 1.20 | 132.0448 m/z | 2-Phenylglycine | C8H9NO2 | 0.8 | 0.045 | 1.2 |  |  |  |
|  | 3.01 | 261.0073 m/z | Homovanillic acid sulfate | C9H10O7S | 7.8 | 0.002 | 21 |  |  |  |
|  | 6.53 | 186.0767 m/z | 2-Keto-6-acetamidocaproate | C8H13NO4 | 1.1 | 0.002 | -1.7 |  |  |  |
|  | 1.34 | 227.1279 m/z | Dibutyl malate | C12H22O5 | 1.7 | 0.002 | 2.1 |  |  |  |
|  | 1.99 | 211.0605 m/z | Propyl gallate | C10H12O5 | 0.4 | 0.03 | 1.8 |  |  |  |
|  | 8.29 | 611.1628 m/z | Aromadendrin 3,7-diglucoside | C27H32O16 | 2 | 0.002 | 13.9 |  |  |  |
|  | 3.45 | 211.0616 m/z | 3-Hydroxy-4-methoxyphenyllactic acid | C10H12O5 | 0.5 | 0.002 | -1.3 |  |  |  |
|  | 2.76 | 258.9916 m/z | Caffeic acid 3-sulfate | C9H8O7S | 4.7 | 0.002 | 2.3 |  |  |  |
|  | 4.51 | 258.9912 m/z | Caffeic acid 4-sulfate | C9H8O7S | 1.4 | 0.045 | -1.4 |  |  |  |
|  | 7.37 | 449.1086 m/z | Phloretin 2'-O-glucuronide | C21H22O11 | 1.3 | 0.002 | 3.4 |  |  |  |
|  | 3.27 | 230.9974 m/z | Hydroxymethoxyphenylcarboxylic acid-O-sulphate | C8H10O7S | 9.2 | 0.065 | 1.3 |  |  |  |
|  | 2.31 | 239.9968 m/z | Indole-3-carboxilic acid-O-sulphate | C9H7NO5S | 4.9 | 0.002 | 1.9 |  |  |  |
|  | 3.04 | 169.0145 m/z | (Z)-But-1-ene-1,2,4-tricarboxylate | C7H8O6 | 0.5 | 0.011 | -1.5 |  |  |  |
|  |  |  | 1636 Unknowns |  |  |  |  |  |  |  |
| **Both WT and Meprin βKO** | 1.26 | 175.0243 m/z | D-Glucuronic acid | C6H10O7 | 2.8 | 0.03 | 1.4 | 2.6 | 0.03 | 1.2 |
|  | 1.71 | 263.0221 m/z | 3-Methoxy-4-hydroxyphenylethyleneglycol sulfate | C9H12O7S | 2.9 | 0.045 | 1.8 | 2.9 | 0.247 | -1.2 |
|  | 1.85 | 245.0126 m/z | 3-Methoxy-4-hydroxyphenylethyleneglycol sulfate | C9H12O7S | 11.7 | 0.045 | 1.5 | 10.3 | 0.931 | 1 |
|  | 12.29 | 195.0509 m/z | Galactonic acid | C6H12O7 | 3.8 | 0.003 | -2.1 | 4.2 | 0.177 | -2.1 |
|  | 3.51 | 204.0663 m/z | Indolelactic acid | C11H11NO3 | 4.6 | 0.065 | -1.5 | 5.8 | 0.082 | -1.5 |
|  | 1.18 | 212.0032 m/z | Indoxyl sulfate | C8H7NO4S | 10.6 | 0.045 | 1.2 | 6.7 | 0.792 | 1.1 |
|  | 3.66 | 178.0510 m/z | Hippuric acid | C9H9NO3 | 9 | 0.002 | 1.5 | 7.2 | 0.082 | 1.3 |
|  | 2.89 | 172.0976 m/z | Isovalerylalanine | C8H15NO3 | 4.7 | 0.03 | -1.5 | 7.6 | 0.052 | -3.7 |
|  | 1.02 | 174.0555 m/z | Phenylacetylglycine | C10H11NO3 | 0.4 | 0.002 | -3 | 0.4 | 0.004 | -2.6 |
|  | 2.99 | 206.0818 m/z | Phenylpropionylglycine | C11H13NO3 | 5 | 0.011 | -2.2 | 3.8 | 0.082 | -1.4 |
|  | 5.42 | 174.0764 m/z | N-Carboxyethyl-g-aminobutyric acid | C7H13NO4 | 2 | 0.002 | -4.8 | 1.8 | 0.03 | -1.8 |
|  | 4.65 | 128.0346 m/z | 1-Pyrroline-4-hydroxy-2-carboxylate | C5H7NO3 | 2.6 | 0.002 | -7.7 | 2.1 | 0.017 | -2.8 |
|  | 7.26 | 160.0399 m/z | Indole-3-carboxylic acid | C9H7NO2 | 2.7 | 0.002 | -4.8 | 2.5 | 0.004 | -3 |
|  | 3.64 | 187.0068 m/z | p-Cresol sulfate | C7H8O4S | 3.9 | 0.002 | 2.1 | 2.6 | 0.247 | 1.2 |
|  | 3.87 | 261.0072 m/z | Homovanillic acid sulfate | C9H10O7S | 3.5 | 0.045 | -1.1 | 5 | 0.126 | 1.1 |
|  | 2.54 | 172.0976 m/z | N-Acetylleucine | C8H15NO3 | 6.4 | 0.006 | 2.7 | 5.2 | 0.329 | 1.3 |
|  | 1.38 | 350.2084 n | 20-oxo-leukotriene B4 | C20H30O5 | 1.7 | 0.003 | 2.6 | 1.8 | 0.009 | 2.3 |
|  | 2.58 | 186.1128 m/z | N-Heptanoylglycine | C9H17NO3 | 4.4 | 0.006 | -2.6 | 6.7 | 0.017 | -3.9 |
|  | 4.39 | 243.0616 m/z | Ng-L-Glutamyl-L-aspartic acid | C9H14N2O7 | 1.3 | 0.045 | -1.4 | 2.3 | 0.03 | -1.5 |
|  | 1.10 | 269.0450 m/z | Porric acid B | C15H12O6 | 3.5 | 0.002 | -3.4 | 3.3 | 0.052 | -1.6 |
|  | 2.95 | 123.0445 m/z | 2-Acetyl-3-methylfuran | C7H8O2 | 1.8 | 0.002 | 1.4 | 1.5 | 0.03 | 1.1 |
|  | 5.85 | 387.2007 m/z | 9,13-Dihydroxy-4-megastigmen-3-one 9-glucoside | C19H32O8 | 1.7 | 0.002 | -19.1 | 1.5 | 0.004 | -3.5 |
|  | 1.12 | 349.0016 m/z | Apigenin 7-sulfate | C15H10O8S | 2.3 | 0.03 | -1.6 | 2.3 | 0.052 | -1.4 |
|  | 2.62 | 349.0019 m/z | Apigenin 7-sulfate | C15H10O8S | 1.7 | 0.03 | -1.5 | 2.1 | 0.052 | 1.3 |
|  | 8.83 | 202.0716 m/z | Avenic acid B | C8H15NO6 | 1.5 | 0.002 | -5.8 | 1.5 | 0.009 | -1.7 |
|  | 14.16 | 351.0566 m/z | a-L-threo-4-Hex-4-enopyranuronosyl-D-galacturonic acid | C12H16O12 | 2.4 | 0.003 | -22.5 | 3.3 | 0.052 | -4.3 |
|  | 6.23 | 387.1653 m/z | beta-D-Glucopyranosyl-11-hydroxyjasmonic acid | C18H28O9 | 2.8 | 0.03 | -2.1 | 2.4 | 0.931 | 1.2 |
|  | 6.96 | 559.1129 m/z | Cicerin 7-(6-malonylglucoside) | C26H26O15 | 4 | 0.006 | 5.8 | 1.4 | 0.004 | 1.8 |
|  | 7.41 | 445.0772 m/z | Genistein 5-O-glucuronide | C21H18O11 | 2.6 | 0.03 | 1.8 | 3.3 | 0.004 | 1.6 |
|  | 1.14 | 167.0706 m/z | 1,3,5-Trimethoxybenzene | C9H12O3 | 2.1 | 0.002 | -3.1 | 1.5 | 0.009 | -1.6 |
|  | 1.26 | 254.9813 m/z | Ascorbic acid-2-sulfate | C6H8O9S | 7 | 0.03 | 1.4 | 6.7 | 0.082 | 1.1 |
|  | 7.12 | 417.1184 m/z | Fenoprofen glucuronide | C21H22O9 | 6.7 | 0.011 | 1.6 | 8.3 | 0.017 | 1.4 |
|  |  |  | 674 Unknowns |  |  |  |  |  |  |  |
